# Toxin production spontaneously becomes regulated by local cell density in evolving bacterial populations

**DOI:** 10.1371/journal.pcbi.1007333

**Published:** 2019-08-30

**Authors:** Hilje M. Doekes, Rob J. de Boer, Rutger Hermsen

**Affiliations:** Theoretical Biology, Department of Biology, Utrecht University, Utrecht, the Netherlands; Memorial Sloan Kettering Cancer Center, UNITED STATES

## Abstract

The production of anticompetitor toxins is widespread among bacteria. Because production of such toxins is costly, it is typically regulated. In particular, many toxins are produced only when the local cell density is high. It is unclear which selection pressures shaped the evolution of density-dependent regulation of toxin production. Here, we study the evolution of toxin production, resistance and the response to a cell-density cue in a model of an evolving bacterial population with spatial structure. We present results for two growth regimes: (i) an undisturbed, fixed habitat in which only small fluctuations of cell density occur, and (ii) a serial-transfer regime with large fluctuations in cell density. We find that density-dependent toxin production can evolve under both regimes. However, the selection pressures driving the evolution of regulation differ. In the fixed habitat, regulation evolves because it allows cells to produce toxin only when opportunities for reproduction are highly limited (because of a high local cell density), and the effective fitness costs of toxin production are hence low. Under serial transfers, regulation evolves because it allows cells to switch from a fast-growing non-toxic phenotype when colonising a new habitat, to a slower-growing competitive toxic phenotype when the cell density increases. Colonies of such regulating cells rapidly expand into unoccupied space because their edges consist of fast-growing, non-toxin-producing cells, but are also combative because cells at the interfaces with competing colonies do produce toxin. Because under the two growth regimes different types of regulation evolve, our results underscore the importance of growth conditions in the evolution of social behaviour in bacteria.

## Author summary

Bacteria live in microbial communities, in which they compete with many other microbes for nutrients and space. In this competitive environment, almost all known bacterial strains produce toxins that impair or kill other bacteria. This chemical warfare is thought to be one of the major factors shaping microbial diversity. Many toxins are produced only if the local density of bacteria is high. To achieve this, bacteria respond to cell-density cues: signalling molecules or other indicators of the presence of other cells. Here, we use a computational model to study the evolution of density-based regulation of toxin production in bacterial populations. We show that such regulation can arise under various growth conditions, and analyse the selection pressures driving its evolution. In particular, we find that if bacteria regularly need to colonise a new habitat, density-based regulation allows them to express a fast-growing, non-toxic phenotype when expanding into uncolonised territory, and a slower-growing, toxin-producing phenotype when competing with other strains. Colonies of regulating cells show a typical structure, with cells of the fast-growing, sensitive phenotype at their expanding edges, and toxin-producing cells in the colony interior and at interfaces between colonies.

## Introduction

Many bacteria produce antimicrobial toxins that impede the growth of competing bacteria or even kill them [1–3]. A wide variety of such toxins has been discovered, ranging from narrow-range bacteriocins to broad-range antimicrobials that may even affect eukaryotic cells [4, 5]. Because producing and secreting toxins is metabolically costly, toxin producing strains have a reduced growth rate compared to non-producers [6–8]. Toxin production is therefore an example of spiteful behaviour: it is costly to the actor and harmful to the recipient [9, 10].

Over the years, the questions how and under what conditions such spiteful production of anticompetitor toxins can evolve have inspired many experimental and theoretical studies [6, 7, 9, 11–14]. These studies showed that the spatial scale over which interactions between bacteria take place is a key determinant of the evolutionary stability of toxin production. Modelling work predicted that toxin production is evolutionarily unstable in homogeneous, well-mixed environments with global interactions (*e.g*., a shaken flask), while stable coexistence between a toxin-producing strain (or killer, K) and sensitive strain (S) can arise in spatially structured environments where interactions are local (*e.g*., agar plates) [7, 11]. Under well-mixed conditions, the K strain is fully outcompeted by resistant (R) cells (which for instance arise from K cells through mutational loss of toxin production but not resistance), because R cells avoid the metabolic costs for toxin production but equally benefit from the killing of S cells by the K strain. In spatially structured environments, however, killing and competition are local processes and hence K cells preferentially benefit from the killing effect of their toxin compared to non-producing cells. The population dynamics then follow local cycles of non-transitive “rock-paper-scissors” interactions: The K strain invades patches of S cells; these K cells are subsequently outcompeted by the R strain; and these R cells are in turn outcompeted by the faster-growing S strain [7]. These local KRS-dynamics cause the emergence of wave-like spatial patterns, in which all three strains (K, R, and S) coexist [11, 15, 16]. These theoretical predictions were confirmed *in vitro* in populations of colicin-producing, -sensitive and -resistant *Escherichia coli* cells growing in flasks or on plates [6, 7], and *in vivo* in enteric bacterial populations in a mouse model [12]. Coexistence of a toxin-producing, -resistant, and -sensitive strain was also found in the more complex environment of a growing biofilm *in vitro* [13], and *in silico* modelling showed that the structure of the biofilm strongly affects the evolution of toxin production [14].

In all studies described above, genes for toxin production and resistance were constitutively expressed. Like many metabolically costly traits, however, toxin production is often tightly regulated [3, 4, 17]. In particular, the expression of many anticompetitor toxins is regulated by cell-density cues: small diffusible molecules that are excreted by bacterial cells, such that their extra-cellular concentration reflects the local density of bacteria (see refs [4, 18, 19] for reviews). Responding to a density cue allows bacteria to express costly genes only when the local cell density is high.

The high prevalence of toxin regulation by density cues raises the question of how such regulation evolved. A common explanation for the regulation of social behaviours by cell-density cues is that the benefits of the regulated social behaviour outweigh the costs only if a sufficient number of cells (the *quorum*) display the behaviour at the same time; the regulation is then also called *quorum sensing* (QS) [20, 21]. This is for instance the case for the cooperative production of some public goods, like siderophores in *Pseudomonas aeruginosa* [22, 23]. For such costly public-good production, both theoretical and experimental work has shown that production of, and response to, a quorum sensing signal can be beneficial, as it allows cells to produce the public good only if the cell density is high and the benefit of coordinated public-good production is hence substantial [23–28].

Whereas the evolution of density-dependent regulation is relatively well-understood in the context of cooperative public goods, its evolution in relation to spiteful toxin production is less well-studied. In a single modelling study, Czárán and Hoekstra (2007) considered whether the evolution of density-dependent toxin production could be explained by similar reasoning as described above for public goods [29]. A key feature of their model is that the toxin was assumed to be effective only if the local density of toxin producing cells exceeded a threshold density, which required toxin producers to cooperate. Furthermore, the model allowed gain and loss mutations of QS signal production, the hypothesis being that a genotype-specific cue that is produced by killer cells only might evolve to inform the killer cells about the local killer cell density. The study found, however, that QS regulation of toxin production was evolutionarily unstable to resistant “cheater” cells that produce the QS signal (and hence induce killer cells to produce toxin) but not the costly toxin [29]. Hence, considering anticompetitor toxin as a type of public good that is cooperatively produced has so far been unsuccessful in explaining density regulation of toxin production, and it remains unclear what selection pressures drive the evolution of toxin regulation by density cues.

Here, we therefore explore different explanations for the evolution of density-dependent toxin production. We use a computational model of evolving, spatially structured bacterial populations that deliberately differs from previous studies. In particular, we do not impose that a minimal quorum of toxin-producing cells is required in order to affect sensitive cells, but instead assume that the effect of toxin increases linearly with its concentration. Also, we focus on cases where toxin production is regulated by density cues that are produced by all cells (including cells that are sensitive to the toxin). For instance, production of the antimicrobial pyocyanin by the common pathogen *Pseudomonas aeruginosa* increases in the presence of peptidoglycan fragments, a general indicator of the local density of gram-positive bacteria [30] (see Discussion for more examples). In such cases, the cue indicates the total cell density rather than the density of killer cells. We obtain results for two growth regimes: (i) a long-term local competition regime, in which the population evolves in a fixed, densely populated habitat, and (ii) a serial-transfer regime in which small, random subsets of the population repeatedly colonise new habitats. We show that density-dependent regulation of toxin production can evolve under both regimes. By characterising the selection pressures shaping the evolution of regulation, we explain how density-dependent toxin production can evolve under various growth regimes.

## Model

We developed a spatially explicit individual-based model of a population of bacteria in which production of anticompetitor toxin, resistance to the toxin, and response to a cell-density cue can evolve. Here, a general overview of the model is given; details of the implementation and analysis are provided in the Methods section.

### Bacteria and their genotypes

**Fig 1:**
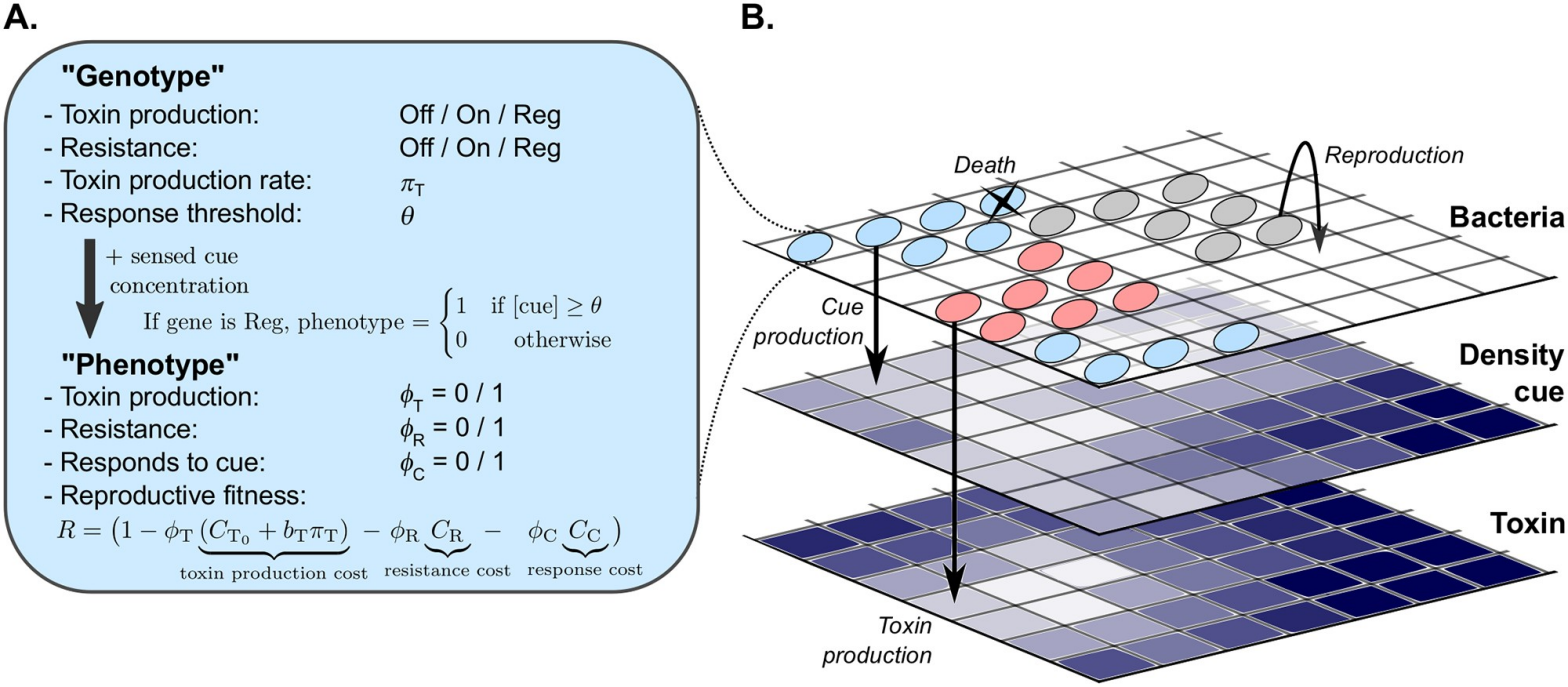
Illustration of the model. (A) The model bacteria’s “genotype” consists of a toxin production “gene”, a resistance “gene”, a toxin production rate *π*_T_ and a response threshold *θ*. Bacteria regulating their toxin production and/or resistance only express these genes if the local concentration of the density cue exceeds the cell’s response threshold. Expression of the toxin, resistance, and response to the cue come at a fitness cost. (B) The model consists of three coupled 2D lattices, which hold the bacteria, the density cue concentration and the toxin concentration. Bacteria locally compete for unoccupied space to reproduce. All cells have a natural death rate. For cells that are not resistant the death rate increases linearly with the local toxin concentration. All bacteria produce the cue, while the toxin is produced only by bacteria that express their toxin gene. The toxin and density cue diffuse and are degraded at fixed rates.

The bacteria in our model live on a square lattice (Fig 1). The model bacteria have several evolvable characteristics, which constitute their “genotype” (Fig 1A). Firstly, they carry a toxin production “gene” and a resistance “gene”, each with three possible alleles: inactive (“Off”), constitutively expressed (“On”), or expressed in response to the density cue (“Regulated”, or “Reg”). We refer to a cell’s toxin production and resistance genotype using a bracket notation: *e.g*., bacteria with genotype “(Reg, On)” regulate their toxin production but constitutively express resistance. Secondly, bacteria that express their toxin gene may differ in their toxin production rate *π*_T_. Lastly, each bacterium has a response threshold value *θ*, which is the cue concentration above which it expresses its regulated genes, if it has any.

### Concentration profiles of the cell-density cue and the toxin

The concentrations of the cell-density cue and the toxin are modelled with partial differential equations describing their local production, degradation, and diffusion. The cue molecule is produced by all cells, while the toxin is only produced by cells expressing the toxin production gene. We consider the dynamics of the cue and toxin to be much faster than the population dynamics of the bacteria, so that the concentration profiles of the cue and toxin at any given time are determined by the current spatial distribution of bacteria [14, 26, 31, 32]. We choose arbitrary units of concentration such that the concentration of the density cue varies between 0 and 1 and the toxin concentration varies between 0 and max(*π*_T_), the largest toxin production rate in the bacterial population (see Methods).

### Dynamics of bacteria

Time in the model progresses in discrete steps. At the beginning of each time step, each bacterium senses the local cue concentration, which together with the cell’s genotype determines the cell’s “phenotype” (Fig 1A). The phenotype is given by three variables: toxin production *ϕ*_T_, resistance *ϕ*_R_, and cue response *ϕ*_C_, which take the value 0 (Not expressed) or 1 (Expressed). If the cell has a regulated gene, the corresponding phenotype value is set to 1 if the cue concentration exceeds the cell’s response threshold value *θ*, and to 0 otherwise. Variable *ϕ*_C_ indicates whether the cell expresses a regulatory response system; it is 1 if the cell has at least one Reg gene, and 0 otherwise.

Note that a regulating cell’s phenotype adapts to the local cue concentration at each simulation time step. An exception to this instantaneous adaptation is made in cells that regulate both their toxin production and resistance (genotype (Reg, Reg)): inspired by the *com*-regulon of *Streptococci* which displays a delay in the expression of the bacteriocins CbpD and LytA relative to the immunity factor ComM [33–35], a delay of *τ*_delay_ time steps is implemented between the expression of resistance and the expression of toxin. This delay prevents cells from killing neighbouring cells that have exactly the same genotype but coincidentally experience a slightly lower cue concentration and therefore do not (yet) express resistance.

Reproduction and cell death depend on a cell’s phenotype. The death rate of sensitive bacteria increases linearly with the local toxin concentration (similar to refs [7, 11, 14]). Importantly, this means that no minimal density (quorum) of toxin producers is required for the toxin to have an effect on sensitive cells (in constrast to [29]). Rather, each toxin producing cell proportionally adds to the killing rate of sensitive cells in the local neighbourhood.

The bacteria locally compete for a growth-limiting resource. To incorporate such local competition, at most one bacterium is allowed to occupy each lattice site. Bacteria surrounding an empty site compete for reproduction based on their respective reproduction rates (which depend on their phenotypes, as described below). When a cell reproduces, the daughter cell inherits the parent’s genotype, except that with small probability mutations are introduced.

### Fitness costs

Toxin production and resistance are metabolically expensive [6–8]. Being able to respond to the density cue requires the production of receptors and a signal transduction pathway, and therefore likely also bears a metabolic cost. We incorporate these metabolic costs by reducing the reproduction rate of cells expressing these phenotypes. The costs for resistance and the ability to respond to the cue are constant, while the cost for toxin production increases linearly with the cell’s toxin production rate *π*_T_. Note that cells that regulate a gene always pay a cost for being able to respond to the cue, but in return may avoid the costs of toxin production and resistance when the density cue concentration is below their response threshold *θ*.

## Results

### Evolution of toxin regulation in a fixed, densely populated habitat

We first considered a bacterial population growing in a fixed habitat without external perturbations, by running the model on an undisturbed simulation lattice. Because we aimed to investigate the evolutionary potential of the production of anticompetitor toxin and its density-dependent regulation in general, rather than to model a specific strain of bacteria, we explored possible evolutionary outcomes of the model by performing a parameter sweep over the six defining parameters of our model: the spatial range of the density cue *L*_cue_, the spatial range of the toxin *L*_tox_, $R_{0}^{-1}$ of the bacteria (where *R*_0_ is the maximal expected number of daughter cells produced per bacterial life time), the scaled toxin production cost ${\hat{b}}_{T}$, the resistance cost *C*_R_, and the cue response cost *C*_C_ (see S1 Text for the derivation of these parameters). We performed 2000 simulations with random parameter settings uniformly sampled from broad parameter ranges (see Methods, Table 1). For each simulation, we then calculated the mean abundance of each genotype and each phenotype after an evolutionary steady state was reached. Based on this evolved population composition, the simulations were classified into four categories (S1 Fig).

**Table 1:** Model parameters.

**Varied in parameter sweep**		
*Parameter*	*Description*	*Range*
$L_{\text{cue}}=2\sqrt{\frac{D_{\text{cue}}}{d_{\text{cue}}}}$	Characteristic length scale of the cell-density cue concentration profile (lattice sites)	[2, 20]
$L_{\text{tox}}=2\sqrt{\frac{D_{\text{tox}}}{d_{\text{tox}}}}$	Characteristic length scale of the toxin concentration profile (lattice sites)	[2, 38]
$R_{0}^{-1}=\frac{\delta }{\gamma }$	$\frac{1}{R_{0}}$ of the bacteria, where *R*_0_ is the maximal expected number of offspring per bacterial life time (dimensionless)	[0, 0.5]
${\hat{b}}_{T}=\frac{\gamma d_{\text{tox}}}{\delta_{\text{tox}}}b_{T}$	Scaled slope of toxin production cost function (dimensionless)	[0.01, 0.8]
*C*_R_	Cost of resistance (dimensionless)	[0.01, 0.25]
*C*_C_	Cost of responding to the density cue (dimensionless)	[0.01, 0.1]
**Fixed**		
*Parameter*	*Description*	*Value*
$C_{T_{0}}$	Offset of toxin production cost function	0.01
*τ*_delay_	Delay between expression of resistance and toxin production in (Reg, Reg)-cells	50 time steps
*μ*_gain_	Probability of a gain mutation upon reproduction	5 ⋅ 10^−5^
*μ*_loss_	Probability of a loss mutation upon reproduction	5 ⋅ 10^−4^
*μ*	Probability that a mutation occurs in response threshold *θ* or toxin production rate *π*_T_ upon reproduction	5 ⋅ 10^−4^
*p*_largemut_	Probability that a mutation in *θ* or *π*_T_ yields a random value	10^−3^
*σ*_*μ*_	Maximum size of a mutation in *θ* or *π*_T_ otherwise	0.05

**Fig 2:**
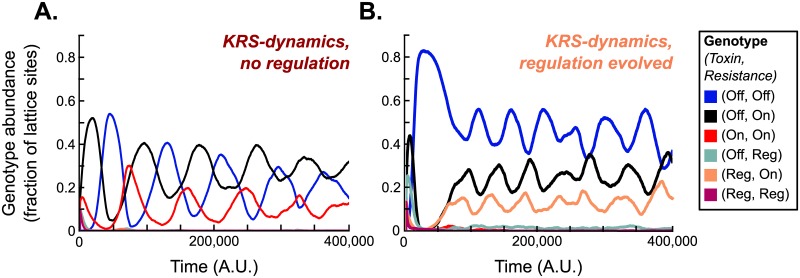
Types of KRS-systems that evolved in a fixed, densely populated habitat. Simulations were initialised with bacteria with random genotypes, and then run until evolutionary steady state was reached. Out of the 2000 simulations in the parameter sweep, 228 resulted in a KRS-system. (A) In 206 runs killer cells (genotype (On, On)), resistant cells (genotype (Off, On)) and sensitive cells (genotype (Off, Off)) coexisted, but no regulation evolved. (B) In 22 runs regulation did evolve, and in most of these (17 runs) coexistence was found between cells that regulate their toxin production but constitutively expresses resistance (genotype (Reg, On)), non-regulating resistant cells (genotype (Off, On)), and sensitive cells (genotype (Off, Off)). Parameter values for the example runs shown here are: (A) *L*_cue_ = 3.7, *L*_tox_ = 16.5, $R_{0}^{-1}=0.1$, ${\hat{b}}_{T}=0.045$, *C*_R_ = 0.12, and *C*_C_ = 0.07; (B) *L*_cue_ = 6, *L*_tox_ = 6, $R_{0}^{-1}=0.125$, ${\hat{b}}_{T}=0.32$, *C*_R_ = 0.1, and *C*_C_ = 0.02.

In 1737 of the 2000 runs, the sensitive genotype (Off, Off) fixed in the population, indicating that most parameter conditions were unfavourable to the evolution of toxin production. In 228 simulations, at least one toxin producing genotype, sensitive genotype, and resistant genotype were found, hence yielding a KRS-system. Most of these evolved KRS-systems consisted of non-regulating killers (genotype (On, On)), non-regulating resistant cells (genotype (Off, On)), and sensitive cells (genotype (Off, Off)) (Fig 2A), reproducing the KRS-dynamics observed in earlier studies [7, 11, 15, 16]. In 22 of the 228 simulations yielding KRS-dynamics, however, at least one regulating genotype was selected. In a clear majority of these (17 runs), a single regulating genotype was found: cells that regulate their toxin production, but constitutively express resistance (genotype (Reg, On)). These regulating cells coexisted with sensitive cells (genotype (Off, Off)) and resistant cells (genotype (Off, On)) (Fig 2B). Lastly, 35 simulations did not result in fixation of sensitives or a KRS-system and were classified as “other”. In none of these simulations regulation evolved, and they were therefore not further considered.

#### Regulation of toxin production can evolve when regulation costs are low, the density cue is short-ranged, and toxin production is costly

**Fig 3:**
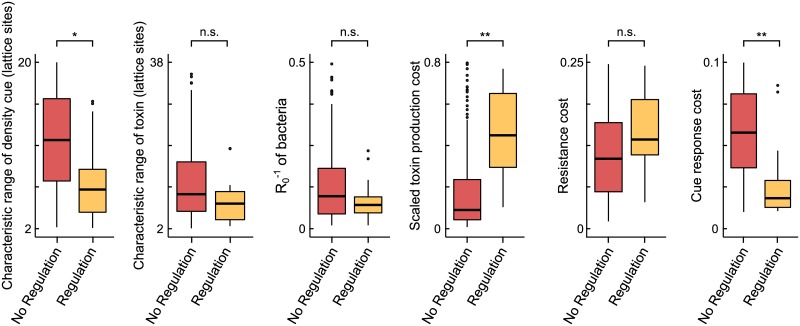
Parameter conditions for the evolution of regulation. The distribution of parameter values for simulations that yielded KRS-dynamics without regulation (*n* = 206) and those that yielded KRS-dynamics with regulation (*n* = 22). In simulations that resulted in the evolution of regulation, the spatial range of the cue and the response costs were lower, while the toxin production costs were higher. Results of 2-sided *t*-tests with Bonferroni-correction for multiple testing: **: *p* < 10^−6^, *: *p* < 10^−3^, n.s.: not significant.

To determine which parameter combinations favour the evolution of regulation, we compared the parameter sets that resulted in KRS-dynamics without regulation (*n* = 206) to those that resulted in KRS-dynamics with regulation (*n* = 22) (Fig 3). Unsurprisingly, in simulations in which regulation evolved, the cost of regulation was typically much lower. Regulation also evolved more readily when toxin production was costly and when the spatial range of the cell-density cue was limited. These conditions seem reasonable: the potential benefits of regulation are largest when it controls a costly behaviour, and a short-ranged cue contains more information about the current local environment than a longer-ranged cue. Since competition in the model occurs over short spatial ranges, responding to a short-ranged cue allows bacteria to quickly adapt to changes in their immediate competition environment.

#### Cells that regulate their toxin production occupy the killer niche in KRS-dynamics

**Fig 4:**
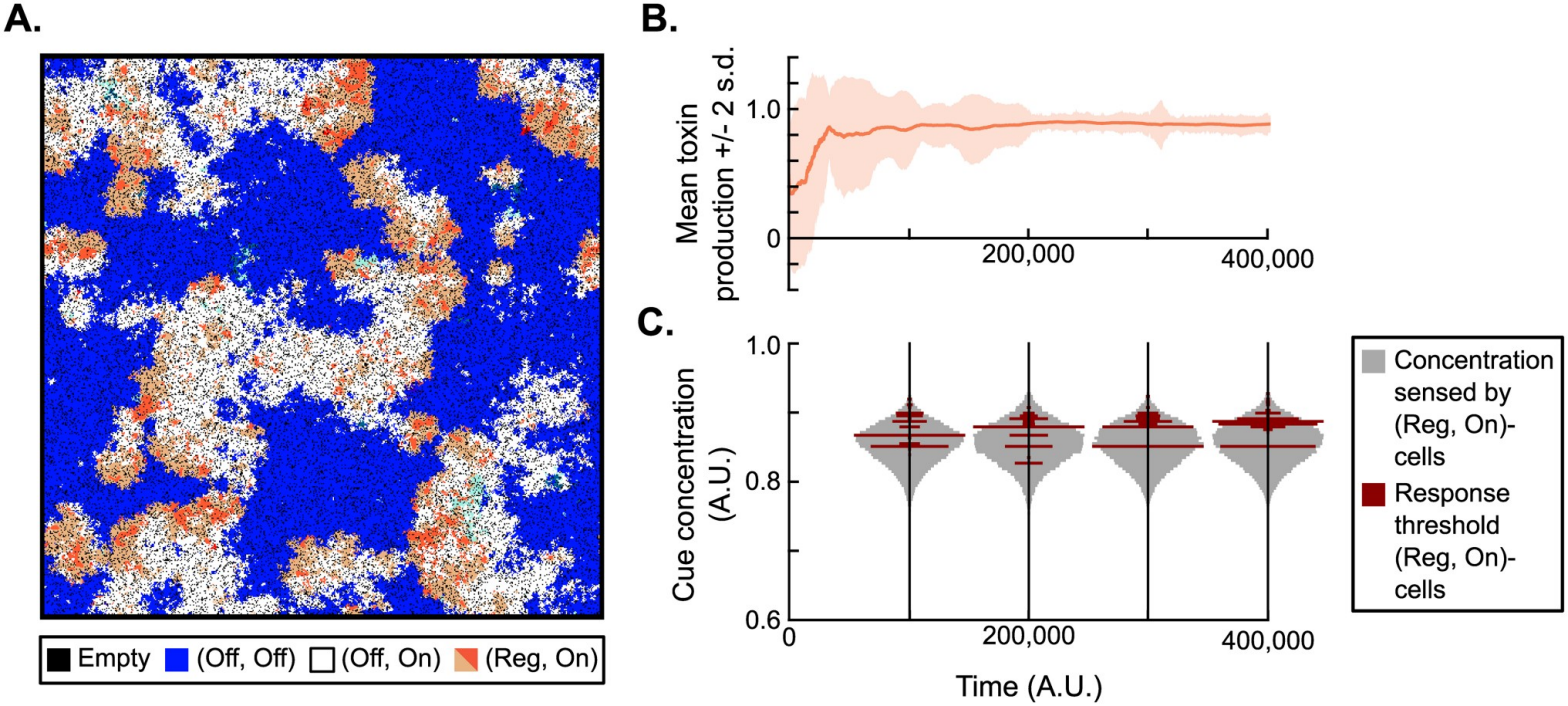
Model dynamics of a run in which density-dependent toxin regulation evolved. (A) Snapshot of the simulation lattice. KRS-dynamics emerge with sensitive cells (genotype (Off, Off), blue), resistant cells (genotype (Off, On), white) and regulating killer cells (genotype (Reg, On), orange). The latter switch between two phenotypes: toxin producing (dark orange) and resistant (light orange). See also S1 Video. (B) Toxin production rate in the (Reg, On)-cells over time. Cells were initialised with a toxin production rate sampled at random between 0 and 1. Over time, a mean value of *π*_T_ ≈ 0.8 is selected. (C) Distribution of response threshold values in (Reg, On)-cells over time, plotted against a background distribution of the cue concentration sensed by these (Reg, On)-cells. Response threshold values around *θ* = 0.875 are selected. The selected response threshold values tend to be higher than the median cue concentration sensed by regulating cells, indicating that at any time only a minority of cells produces toxin.

To better understand how density-dependent toxin regulation evolved in our model, we studied the example run of Fig 2B in more detail. The evolving population displayed KRS-dynamics, with regulating killer cells (genotype (Reg, On)) invading patches of sensitive cells (genotype (Off, Off)), constitutively resistant cells (genotype (Off, On)) invading patches of regulating killer cells, and sensitive cells invading patches of resistant cells (Fig 4A, S1 Video). The (Reg, On)-cells hence acted as the killer in these KRS-dynamics, replacing the constitutive killers (genotype (On, On)) found in non-regulating KRS-systems.

When the evolved (Reg, On)-cells express their toxin gene they produce a considerable amount of toxin (*π*_T_ ≈ 0.8, Fig 4B), but due to regulation they do so only when they sense a high cue concentration. This regulation is governed by the evolved response threshold *θ*, the cue concentration above which regulated genes are expressed. To interpret the observed response threshold values, we compared them to the distribution of cue concentrations that (Reg, On)-cells sense (Fig 4C). Most response threshold values were greater than the median cue concentration observed, indicating that evolved (Reg, On)-cells are more often in their inactive resistant state than in their active toxin-producing state.

To make sure that we do not base our conclusions on contingencies in a particular example run, we performed ten replicate runs with the same parameter settings, and found that the results were highly reproducible (S2 Fig).

#### Regulation decreases the effective fitness cost of toxin production

What selection pressures drive the observed evolution of density-dependent toxin regulation? In the cyclic KRS-system, two main factors determine the success of toxin-producing cells: their competitive advantage over sensitive cells and their disadvantage to resistant cells. To quantify the effect of regulation on these competitive (dis)advantages, we performed controlled invasion experiments comparing the invasion dynamics of the evolved (Reg, On)-cells to non-regulating (On, On)-cells evolved under the same parameter conditions but in a simulation where regulation was disabled (S3A Fig). First, we directly competed the regulating (Reg, On)-strain with the (On, On)-strain, and found that the regulating killer strain invades and eventually fully displaces the constitutive killer strain, as expected. Next, we calculated the speed at which both killer strains invade a sensitive population, and the speed at which they are invaded by a resistant strain (S3B Fig). Surprisingly, compared to the (On, On)-strain, the (Reg, On)-strain both invaded a sensitive population faster **and** was invaded more slowly by the resistant strain (S3C Fig). Cells that regulate their toxin production hence have an advantage over non-regulating killer cells both in their invasion of new patches of sensitives, and in their competition with resistant cells.

The difference in the invasion speed into sensitive patches is explained by a difference in mean toxin production: the evolved (Reg, On)-cells on average produce more toxin per cell (mean long-term toxin production rate: 0.20) than the (On, On)-cells that evolved under the same conditions if regulation was excluded (mean long-term toxin production rate: 0.13). Naively, a higher mean toxin production rate should result in higher fitness costs. However, the increased competitiveness against resistant cells and the results of the direct competition between the two killer strains suggest that the regulating cells actually have a higher effective reproductive fitness than the constitutive killers. Here, the information conferred by the density cue comes into play. Remember that the metabolic costs of toxin production lead to a reduction in the producing cell’s reproduction rate. However, because reproduction can only occur if empty lattice sites are available, the *effective* fitness costs of a reduction in reproduction rate also depend on the cell’s environment and social neighbourhood. If several lattice sites in the cell’s neighbourhood are unoccupied, the local competition for reproduction is a major determinant of the cell’s fitness and the effective fitness costs resulting from the metabolic costs of toxin production are high. On the other hand, if no neighbouring lattice sites are vacant, the cell has no opportunity to reproduce and the effective fitness costs vanish. By exploiting the cue, the evolved (Reg, On)-cells produce toxin only when none or at most one of their neighbouring sites is empty, and never do so when more than two neighbouring lattice sites are empty (S4 Fig). At the wavefront where the (Reg, On)-cells compete with sensitive (Off, Off)-cells, the produced toxin frees up lattice sites by killing sensitive cells, causing a drop in the density cue concentration, which leads to the expression of the faster-reproducing, non-toxic phenotype in the (Reg, On)-cells benefitting from these available sites (which either produced the toxin themselves or profit from toxin production by closely related neighbouring (Reg, On)-cells). Regulation thus allows cells to produce toxin when reproduction opportunities are scarce and the effective fitness costs of production are hence low, and to exhibit a faster-replicating resistant phenotype when more space is available and hence competition for rapid reproduction is stronger.

#### Regulation works only if the density cue is sufficiently reliable and phenotypic adaptation is sufficiently fast

The evolved regulation mechanism described above requires that the cue concentration conveys detailed information about the environment. The evolved system might therefore be vulnerable to disturbances in the cue, *e.g*., caused by stochasticity in production of the cue molecule, its diffusion and degradation, or in the response pathway. To test this hypothesis, we performed simulations in which at each time step independent Gaussian noise was added to the cue concentration at each lattice site. Regulation still evolved in 4 out of 5 replicate runs if a moderate noise level was used (standard deviation of noise was *σ*_noise_ = 0.025, which is comparable to the change in local cue concentration experienced if one of the eight direct neighbouring cells is removed). At an increased noise level (*σ*_noise_ = 0.05), however, only 2 out of 5 replicate runs showed evolution of regulation, and at an even higher noise level (*σ*_noise_ = 0.1) regulation evolved once in 5 replicate runs. Hence, regulation is effective only if the cue concentration is a sufficiently precise predictor of the current local density.

In the model, when the cue concentration changes, regulating cells adjust their phenotype instantaneously. In reality, such a phenotypic switch takes time [36, 37]. To investigate how such a lag affects our results, we introduced a lag time between sensing a change and altering the phenotype. Regulation still evolved when a relatively short lag times of 5 simulation time steps was used, which is equivalent to 50% of the minimal doubling time of the bacteria (S5 Fig). When the lag time was longer, regulation no longer evolved (S5 Fig), indicating that the evolved regulation mechanism requires cells to be able to adjust their phenotype relatively fast. This result seems intuitive: a regulation mechanism that relies on cells reverting to a fast-growing, non-toxin-producing phenotype when locally empty sites are detected can only be effective if this reversion happens faster than the recolonisation of these empty sites.

### Evolution of toxin regulation under a serial-transfer regime

So far, we have considered model bacteria living in a fixed, undisturbed habitat. Natural growth conditions, however, tend to vary substantially over space and time, and such variations in growth conditions may cause large fluctuations in cell density. To examine how externally induced density fluctuations affect the evolution of density-dependent toxin regulation, we simulated serial transfers: a procedure, well-known from experimental evolution studies, in which a small sample of the population is regularly transferred to fresh medium [38–42]. The population dynamics were simulated as before, except that periodically the simulation was paused, a random sample of cells was taken from the population, and these founder cells were randomly placed on a new simulation lattice (“fresh medium”). These serial transfers were continued for many cycles to allow the system to approach evolutionary steady state.

#### Regulation evolves more frequently under serial transfers than in a fixed habitat

To explore the model’s behaviour under the serial-transfer regime, we simulated serial transfers for the same 2000 parameter conditions used in the fixed-habitat parameter sweep. The evolutionary outcome of simulations was again classified based on genotype and phenotype abundances at evolutionary steady state (S6A Fig). Now, 1894 out of the 2000 parameter combinations resulted in the fixation of sensitives, an even larger fraction than in the fixed-habitat case. This makes sense: if cells regularly have to colonise a new, unpopulated environment, selection is expected to favour the fast-replicating sensitive genotype. Toxin production and resistance did evolve in 86 simulations, which were hence classified as “KRS”. The 20 remaining simulations of the parameter sweep were classified as “other”, and were further disregarded.

Out of the 86 simulations that yielded a KRS-system, regulation evolved in a majority of 68 cases (S6A Fig). Hence, regulation evolved more readily under the serial-transfer regime than in the fixed habitat (in which only 22 parameter combinations out of 206 simulations yielding KRS-dynamics resulted in the evolution of regulation). Compared to the simulations that yielded a KRS-system without regulation (*n* = 18), the simulations in which regulation did evolve (*n* = 68) had relatively high toxin production and resistance costs, and low response costs (S6B Fig), consistent with the observations in the fixed habitat (*c.f*., Fig 3).

#### Under serial transfers, bacteria are selected for fast colony expansion

**Fig 5:**
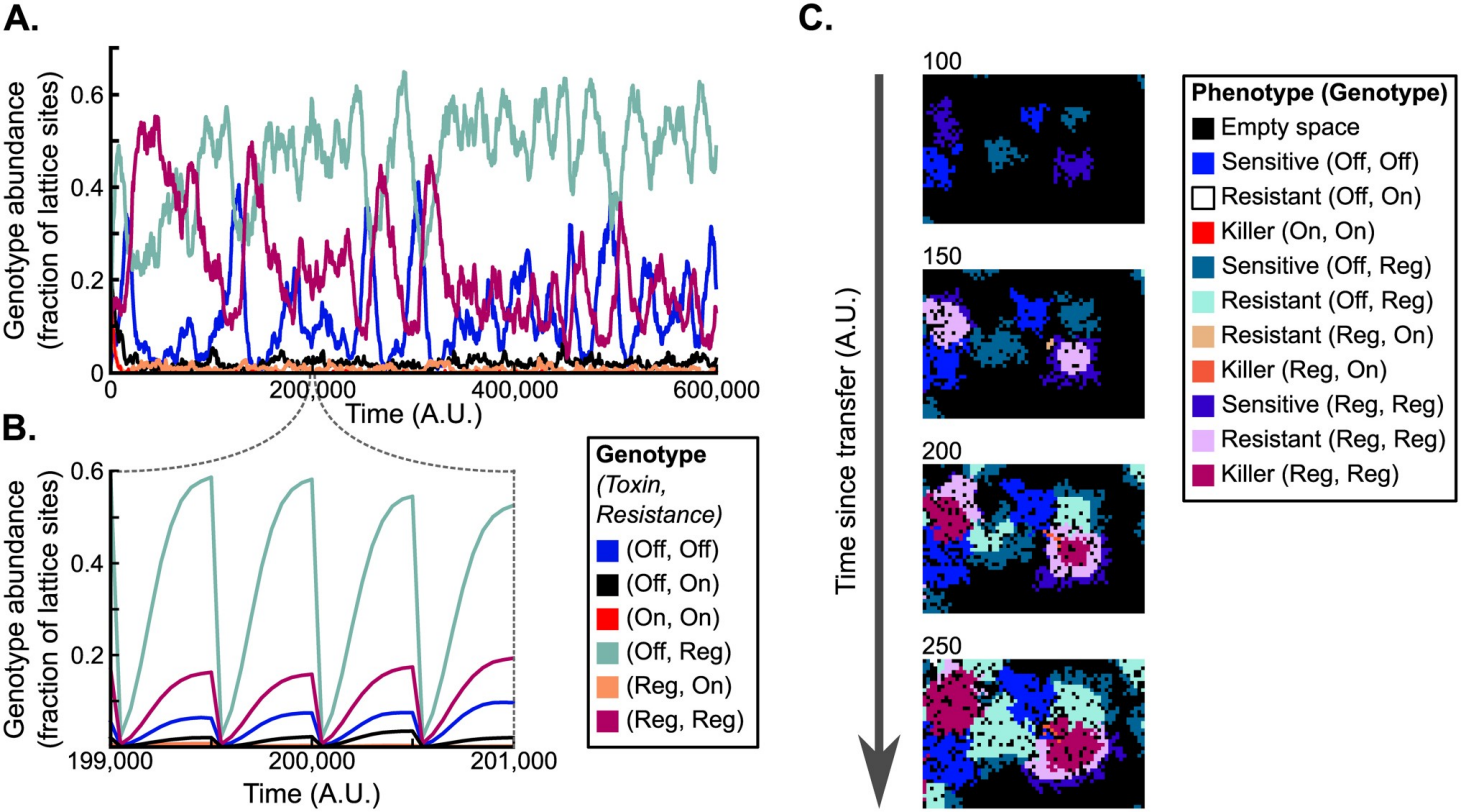
Model dynamics under a serial-transfer regime. The simulation was initialised with cells with random genotypes. Every 500 time steps, a random sample of 1000 cells from the current population was transferred to a new, empty lattice (“fresh medium”). (A, B) Abundance of genotypes over time on long (panel A) and short (panel B) time scales. Since the number of cells varies greatly within each transfer cycle, in panel A only the genotype abundances observed at the end of each cycle are plotted. The evolved population mainly consists of three genotypes: sensitives (genotype (Off, Off)), regulating resistants (genotype (Off, Reg)), and regulating killers, that also regulate their resistance (genotype (Reg, Reg)). (C) Snapshots of a small part of the simulation lattice showing colony growth between two transfers. Early on, (Off, Off)-, (Off, Reg)- and (Reg, Reg)-cells all express the sensitive phenotype. As the size of the colonies increases, the phenotype of cells in the interior of (Off, Reg)- and (Reg, Reg)-colonies switches to resistant, and in the case of (Reg, Reg)-cells after *τ*_delay_ time steps to toxin producing. Cells on colony edges remain sensitive, allowing the colony to grow rapidly. See also S2 Video. Parameter values: *L*_cue_ = 6, *L*_tox_ = 6, $R_{0}^{-1}=0.125$, ${\hat{b}}_{T}=0.072$, *C*_R_ = 0.1, and *C*_C_ = 0.02.

In 62 of the 68 simulations in which regulation evolved, two types of regulating cells were found: (i) (Reg, Reg)-cells that regulate both their toxin production and their resistance, and (ii) (Off, Reg)-cells, that regulate their resistance and do not produce toxin. These cells coexisted with (Off, Off)- and sometimes (Off, On)-cells. A typical example of such dynamics is shown in Fig 5 and S2 Video, and we next consider this example in more detail.

At low cell density, cells with the three dominant genotypes ((Off, Off), (Off, Reg) and (Reg, Reg)) all express a sensitive phenotype. Cells with a sensitive phenotype have low fitness costs and hence a high replication rate. The genotypes (Off, Off), (Off, Reg) and (Reg, Reg) dominated in all ten replicate simulations performed (S7 Fig), indicating that serial transfers robustly select for genotypes capable of growing at a high rate when cell density is low.

Shortly after each transfer into a new, empty, simulation lattice, founder cells of the three evolved genotypes indeed form colonies with a sensitive phenotype (Fig 5C, first panel; S2 and S3 Videos). As the colonies grow, the cue concentration within colonies increases. This causes cells in the interior of (Off, Reg)- and (Reg, Reg)-colonies to switch phenotype and become resistant (Fig 5C, second and third panel; S2 and S3 Videos). After the delay *τ*_delay_ between expression of resistance and toxin production, the (Reg, Reg)-cells furthermore switch to a toxin producing phenotype (Fig 5C, third and fourth panel; S2 and S3 Videos). As a consequence, a ring of non-producing resistant (Reg, Reg)-cells forms that acts as a buffer between the outer layers of sensitives cells and the toxin producing cells in the colony interior. While the phenotype of cells in the interior switches to resistant or toxin producing, the cells at the colony edge retain a sensitive phenotype. From a functional perspective, this again seems reasonable: colonies grow at their edges, and expressing a sensitive phenotype at the colony edge maximises the rate at which a colony expands into unoccupied space. Since cell density is per definition low at the edge of a colony, regulation based on the density cue allows toxin producing and resistant cells to express the fast-replicating sensitive phenotype exactly there where the colony is growing.

#### Regulation allows cells to adjust their phenotype to changing growth conditions

**Fig 6:**
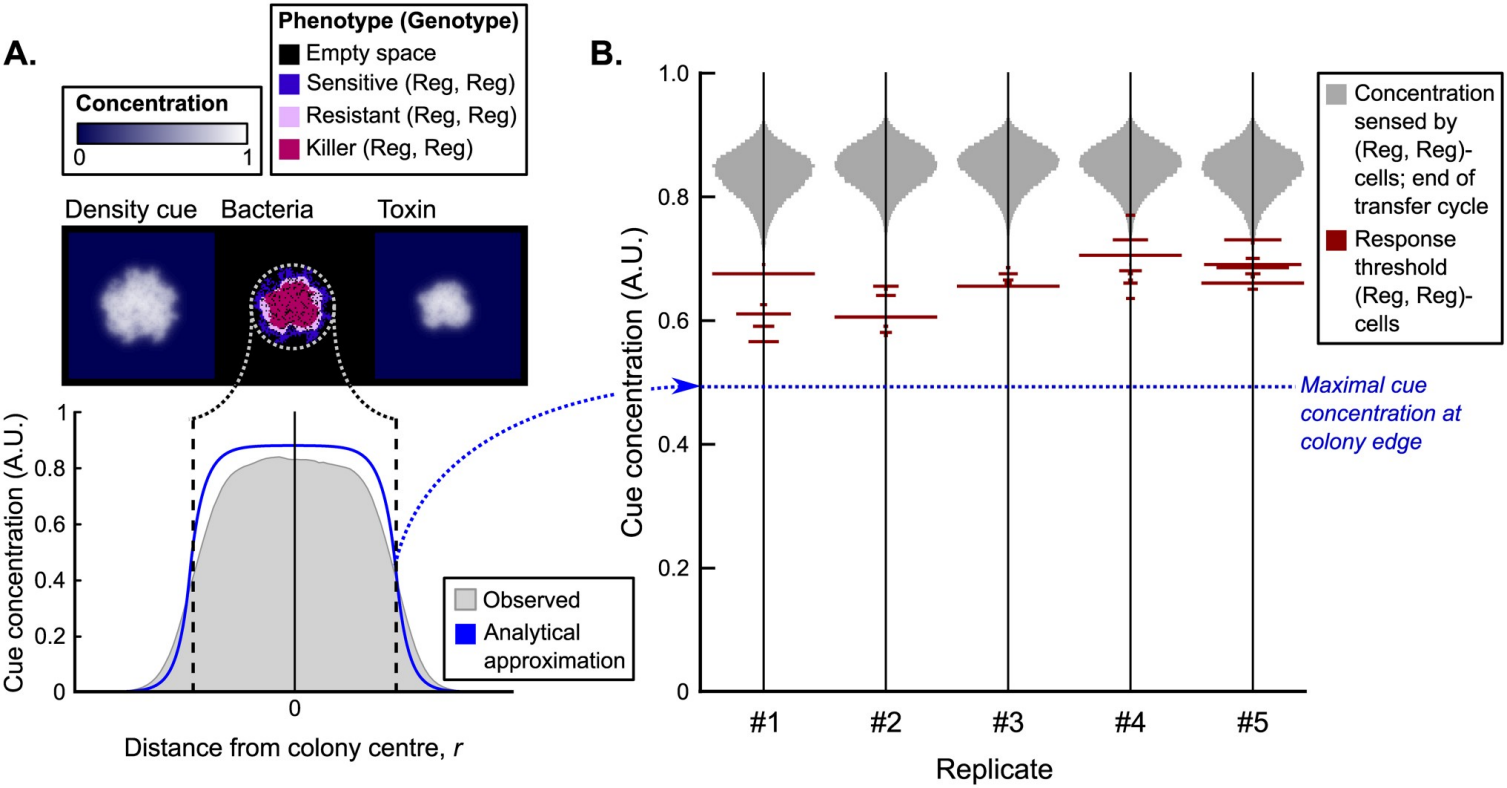
Density cue concentration profile of expanding colonies and the evolved response threshold values of (Reg, Reg)-cells. (A) Colonies were grown from a single (Reg, Reg)-cell to characterise the radial density cue concentration profile of an expanding colony. Measured values from the simulations correspond well to the analytical approximation (see S2 Text). (B) Distribution of the evolved response threshold values in (Reg, Reg)-cells at the end of the simulation (time = 600, 000), plotted against the background of the cue concentration sensed by these cells at the end of a transfer cycle (*i.e*. when the population approaches carrying capacity) for five replicate simulations. The evolved response threshold values vary somewhat between replicates, but are always lower than the cue concentration at local carrying capacity (grey distributions) and higher than the maximum of the cue concentration at the edge of a growing colony. Hence, cells on the colony edge never express their toxin production and resistance genes, while cells in the colony interior and at the interface between colonies (where local cell density is close to carrying capacity) are resistant and do produce toxin.

Regulating cells can maintain a sensitive phenotype at the edge of growing colonies if their response threshold *θ* is larger than the cue concentration at the edge. Approximating the shape of a colony by a circle, we analytically calculated that the cue concentration at the edge of a colony is ≤ 0.49 (S2 Text). This analytical approximation corresponds well to measurements from single-colony simulations (Fig 6A). Although there is some variation between response threshold values of (Reg, Reg)-cells in the replicate runs, all values were well above this lower bound (Fig 6B), showing that the evolved (Reg, Reg)-cells indeed exploit the density cue to form fast-expanding colonies.

Next to the lower bound, the evolved response threshold values are bounded from above by the cue concentrations that cells perceive at the end of a transfer cycle, *i.e*., when the population approaches carrying capacity (Fig 6B). The evolved (Reg, Reg)-cells hence do produce toxin (and consequently pay fitness costs) when the local cell density is high, suggesting that these cells have been selected to exhibit their competitive phenotype (*i.e*., toxin production) when the population locally approaches carrying capacity. Importantly, such high cell densities not only occur in the interior of expanding colonies, but also at the interface between colonies where cells of the two colonies (with potentially different genotypes) compete. At the interfaces between colonies, (Reg, Reg)-cells hence express their toxin producing phenotype (S3 Video). The evolved regulating cells adjust their phenotype to varying growth conditions, exhibiting a sensitive phenotype when replicating into unoccupied space, and expressing toxin production and resistance when in competition with other cells.

#### Over many transfer cycles, the (Reg, Reg)-, (Off, Reg)- and (Off, Off)-cells show KRS-dynamics

So far, we have focussed on explaining the evolution of cells that regulate both their toxin production and resistance. However, a large fraction of the evolved populations is made up of cells that do not produce toxin and regulate their resistance only (Fig 5A and S7 Fig). These (Off, Reg)-cells had response threshold values very similar to those of (Reg, Reg)-cells (S7 Fig), and hence express the same fast-growing sensitive phenotype at the edges of growing colonies, but exhibit a resistant phenotype at the colony interior and where colonies interact (Fig 5C, S2 and S3 Videos).

While the toxin-producing phenotype of (Reg, Reg)-cells provides an advantage when competing with a colony of sensitive (Off, Off)-cells, it confers a disadvantage when competing with a colony of (Off, Reg)-cells. Similarly, the competitive resistant phenotype of (Off, Reg)-cells yields a competitive advantage against (Reg, Reg)-cells, but a disadvantage against (Off, Off)-cells. Hence, within the evolved regulating genotypes we again found cyclic dominance (see S3 Video), and this is reflected in the long term population dynamics (Fig 5A): the number of (Off, Off)-cells, (Off, Reg)-cells and (Reg, Reg)-cells oscillated in a K-R-S order. These KRS oscillations now occur on the time scale of many transfer cycles.

#### The evolution of regulation under serial transfers is highly robust to variations in the transfer regime, cue concentration and phenotype expression timing

Because serial transfers have such a profound impact on the evolution of regulation, we examined how the evolutionary outcome depends on the frequency of transfers and the number of founder cells used to seed the next population (S8 Fig). The evolution of regulation depends on the balance between selection for fast colony growth in sparsely populated environments, and selection for competitive phenotypes in dense environments. When transfers were very frequent, or the number of founder cells was very small, cells were continuously selected for fast growth and the sensitive (Off, Off)-genotype fixed in the population. On the other hand, when transfers were very infrequent or the number of founder cells was very large, we recovered the results found in the absence of serial transfers. In between, however, regulation was found in a wide parameter range: regulating genotypes still evolved when the time between transfers was increased 5-fold compared to the example parameter set of Fig 5, or the number of founder cells was increased by an order of magnitude (S8 Fig).

Regulation also readily evolved when we relaxed the assumption that the time between subsequent transfers is fixed and instead transferred the population with a fixed probability per time step (that is, as a Poisson process, producing an exponential waiting-time distribution) (S9 Fig). Results of these simulations were similar to the results obtained with fixed transfer cycle lengths, indicating that the evolution of regulation is robust against variation in transfer timing.

Furthermore, the evolution of regulation was also robust to variation in the time delay *τ*_delay_ between expression of resistance and toxin production in (Reg, Reg)-cells, with (Reg, Reg)-cells evolving even when *τ*_delay_ = 0 and the ring of non-producing resistant cells in colonies disappears (S10 Fig). Regulation also readily evolved when significant lag times (up to 3 bacterial doubling times) were implemented between the first instance that cells sense a change in cue concentration and the moment these cells change their phenotype accordingly (S11 Fig).

Lastly, the regulatory mechanism that evolved under serial transfers is highly robust to noise in the cue concentration. Even when large Gaussian noise (*σ*_noise_ = 0.1, on cue concentrations varying between 0 and 0.85) was added to the cue concentration at each lattice site at each time step, regulation still evolved (S12 Fig). The regulation mechanism that evolved under the serial-transfer regime is hence more robust to noise in the cue concentration than the mechanism that evolved in a fixed habitat.

#### Spatial structure is crucial for the evolution of toxin production and regulation

So far, we have considered a spatially structured population in which reproduction and competition occur locally. This implementation was chosen because previous work has shown that constitutive toxin production is not evolutionarily stable in well-mixed environments [7, 11] (see Introduction). We therefore did not expect toxin production to evolve in our model in the absence of spatial structure, even if regulation was allowed.

To test this, we repeated all runs of our parameter sweep (both in a fixed habitat and under serial transfers) but now randomised the positions of the bacteria at each time step. We then classified the evolutionary outcome of these simulations in the same way as we analysed the spatially structured simulations (see S1 Fig). As expected, the sensitive genotype (Off, Off) fixed in all 4000 simulation runs. Particularly, even under parameter conditions that did yield (regulating) toxin producing cells in the spatially structured simulations, toxin production did not evolve under well-mixed conditions. This result underscores the crucial importance of spatial structure for the evolution of toxin production, regulated or not.

## Discussion

Using a simulation model, we have shown that the production of anticompetitor toxins can become regulated by a cell-density cue in evolving populations under two different growth regimes: in a fixed habitat, and in serial-transfer cycles. Under both regimes, regulation of toxin production evolves because it allows cells to adjust their investment in toxin production to changes in the local competition and growth opportunities. However, the selection pressures driving the evolution of toxin production at high density, and the resulting types of regulation that evolve, differ between the growth regimes.

In the fixed habitat, regulating killer cells evolved that produce toxin only at very high local cell densities (Fig 4). We showed that these cells use the density cue to produce toxin only if reproduction opportunities are very scarce and the effective fitness costs of toxin production are therefore low (S4 Fig). This type of regulation relies on the fact that, in the model, cells that cannot reproduce due to a lack of empty neighbouring lattice sites can nevertheless produce toxin, at very low or even zero fitness cost. This phenomenon could occur in reality if at low cell density reproduction and toxin production are limited by the same resource(s) (*e.g*., the availability of carbon or nitrogen substrates), while at high cell density reproduction is limited by a different resource that does not limit toxin production (*e.g*., crowding or a lack of substrate not required for toxin production). Interestingly, such conditions have previously been found to stabilise cooperative secretions of swarming-promoting biosurfactants in *Pseudomonas aeruginosa* [43]. Production and secretion of these carbon-rich biosurfactants is regulated by nutrient availability, such that they are only produced when growth is limited by another nutrient than carbon (in this case, the nitrogen source) and the fitness costs of biosurfactant secretion are hence low, a mechanism called *metabolic prudence* by Xavier *et al*., 2011 [43]. Our model hence predicts that such *metabolic prudence* could also promote the evolution of density-dependent toxin regulation in long-term local competition environments by reducing the effective fitness costs of toxin production.

Under the serial transfer regime, we find that the evolution of regulation is dictated by two selection pressures: (i) selection for fast reproduction at the edge of expanding colonies, and (ii) selection for the expression of competitive phenotypes (toxin producing and/or resistant) at the interface between colonies (Figs 5 and 6). The dynamics of single cells founding expanding colonies leads to competition between these clonal colonies, and bacteria are selected for the colony structure that they produce (see S3 Video). After a serial transfer, those colonies that express a sensitive phenotype at their edges expand more rapidly into the newly available empty (or “resource-rich”) space. Regulation allows cells to recognise the expanding edges of their colonies, because the local cell density at colony edges is low, and to thus express a sensitive phenotype at these edges. The selection for fast colony expansion explains why cells are selected to express a sensitive phenotype at the edge of expanding colonies, but does not explain why expression of resistance and/or toxin production at high cell density is favoured. As long as a colony clonally expands without interacting with other colonies, the production of toxins does not confer any benefit. However, since the colony expands at its edges and the spatial range of the toxin is limited, the observed production of toxin in the interior of the colony also does not hamper the fast expansion of the colony. As soon as the expanding colony meets another colony, the situation changes: toxin production then yields a potential benefit in the competition with cells of the other colony (which might be sensitive to the toxin). Regulating cells cannot distinguish between the interior of a single colony or the interface between two colonies, because at both sites the local cell density is high. Responding to high cell density however allows the cells to express their competitive phenotype (toxin production or resistance) when in direct competition with cells of another colony (thus performing “competition sensing” *c.f*., [4]), while expression of the competitive phenotype in the interior of the colony does not slow down the colony’s expansion.

The marked differences between results obtained in the fixed habitat and under serial transfers show that the evolutionary dynamics in the model strongly depend on the growth regime. This is not just true for the model presented here. For instance, in experimentally grown colonies of toxin-producing, resistant, and sensitive *E. coli* strains it was found that populations under range expansion do not always show the coexistence patterns found in a stationary environment [44]. In experimental evolution, it has also long been known that the experimental regime can pose strong selection pressures on evolving populations [45, 46]. Because results obtained in one growth condition often do not generalise to other conditions, it is important to consider multiple regimes in theoretical and experimental evolutionary studies.

The differences between the evolution of regulation in the two growth regimes also warrant the question which results provide the more likely explanation for the observed density-dependent regulation of toxin production in nature. In our model, regulation evolved more frequently in the serial transfer regime than in the fixed habitat (S6A Fig). The regulation that evolved under serial transfers was also more robust to a lag between the change in cue concentration and the switch in phenotype (S5 and S11 Figs), and to noise in the cue concentration (S12 Fig). Lastly, the limitation of toxin production in the fixed habitat to instances where the effective fitness cost is very low (similar to *metabolic prudence*, *c.f*., [43]) can only explain the evolution of density-dependent toxin regulation if specific conditions on the resources limiting reproduction and toxin production are met (see Discussion above). The use of the density cue to recognise the edge of an expanding colony after a serial transfer, however, can favour the evolution of density-dependent toxin regulation as long as bacterial replication is limited by a resource that is present at higher concentration on the edge of a colony than in its interior. This seems to be a fairly general condition. Although “metabolic prudence” might contribute to the evolution of regulation for some toxins, we consider selection for the ability to switch between a fast-growing phenotype when colonising a new environment and a competitive phenotype when competing with other bacteria as the biologically more feasible and general candidate to explain evolution of density-dependent toxin regulation.

A similar switch between a fast-growing phenotype when colonising an environment and slower-growing social phenotypes when cell density is high was recently found in a model of quorum-sensing (QS) regulated cooperative public good production in growing biofilms [47]. In this model by Schluter *et al*. (2016), cells that regulated the production of costly public good through QS were found to outcompete constitutive producer cells, because the regulating cells exhibited a fast-growing, non-producer phenotype during the early stages of biofilm growth, and only switched to public good production when cell density increased. Regulation hence allowed colonies to expand rapidly when cell density was low, and to express a cooperative phenotype when cell density was high. Although this selection for fast colonisation is indeed reminiscent of our results, the selection pressures underlying the social behaviour (public good secretion or toxin production) differ substantially. In the case of public good secretion, Schluter *et al*. show that the QS signal acts as a measure of local relatedness, allowing cells to delay the secretion of public good until they are surrounded by clone mates and the benefit of public good production is high. In the case of toxin production, however, relatedness is a double-edged sword. While toxin production is promoted by high relatedness between toxin producers and those benefitting from the killing, this benefit only arises when non-related sensitive cells are present in the local neighbourhood [48, 49]. In the model presented here, the benefits of the toxin production at high cell density are not explained by high local relatedness, but rather by the presence of (unrelated) sensitive competitors.

A key feature of the density cue considered in this study is that it is produced by all bacteria. The choice of such a “total-density” cue was inspired by many natural examples of regulation by such cues. For instance, expression of the bacteriocin mutacin in the dental bacterium *Streptococcus mutans* is regulated by autoinducer-2 (AI-2) [50, 51], a general quorum sensing molecule that is produced by many species of bacteria as a metabolic byproduct [52, 53]. AI-2 is also involved in the regulation of bacteriocin production in the insect pathogen *Photorhabdus luminescens* [54]. Additionally, in the common pathogen *Pseudomonas aeruginosa* the production of the broad-spectrum antimicrobial pyocyanin is enhanced by the presence of peptidoglycan fragments, which indicates high local density of gram-positive bacteria [30].

In addition to the examples of regulation by total-density cues provided above, a wide variety of density-dependent toxin regulation mechanisms exists. Instead of using a density cue as an indicator for the presence of competitor cells, some bacteria more directly sense the presence of competitors, for instance through cell damage caused by these competitors, and respond with toxin production [55, 56]. Other toxins promote their own production; examples include several colicins [56–58] and the lantibiotics, a large class of bacteriocins produced by Gram-positive bacteria including nisin and subtilin [18, 59]. For these bacteriocins, a modelling study showed that during invasion events, cells that regulate their bacteriocin based on cell density outcompete cells that constitutively express bacteriocin, if the cost of bacteriocin production and the amount of bacteriocin produced are high [60]. Although this study did not consider long-term evolutionary dynamics and did for instance not include resistant, non-toxin-producing strains, its prediction agrees with the conditions that we find for the evolution of regulation (see Fig 3 and S6B Fig).

Notably, the expression of many other toxins is regulated by quorum sensing molecules that seem to be produced specifically for regulation of the toxin [4, 17, 61]. These QS molecules are often produced by toxin producing cells only, and hence act as “killer-specific” cues. A population in which such a killer-specific quorum sensing signal evolves might be prone to social cheating on the signal, *e.g*., by cells that produce the signal but do not produce the toxin, or by cells that cease their signal production and go “under the radar”. Such social signalling cheaters cannot arise for total-density cues, because these are by definition produced by all cells. The evolutionary explanations presented in this work for regulation by total-density cues therefore cannot necessarily be generalised to regulation by killer-specific cues. As far as we are aware, only a single modelling study has been undertaken to examine the co-evolution of toxin production and potentially killer-specific quorum sensing, which found that QS control of toxin production was unstable to social cheating [29] (see also the Introduction). In this model, however, cell density was fixed, and only the local population composition (*e.g*., the fraction of toxin producing bacteria) varied over time. In the model presented here, we have seen that large fluctuations in cell density drastically change the evolution of social behaviours associated with cell density, and might favour the evolution of regulation. Studying the evolution of toxin regulation by killer-specific cues under serial transfers is therefore an interesting and promising direction for future research.

Testing whether or not bacteria employ the types of regulation we identified would require the careful monitoring of the temporal dynamics of toxin production, resistance and cell division in bacterial colonies expanding after serial transfers or growing on a plate at high density, preferably at single-cell level (as *e.g*., done by Mavridou *et al*. [56]). Under serial transfers, the model predicts that bacterial colonies should consist of toxin-producing cells at the interior of the colony, and sensitive cells on the edge of the colony. Furthermore, these sensitive cells at the edge should switch to a toxin-producing phenotype when encountering another colony. These predictions can be tested by following toxin production at the single-cell level in growing bacterial colonies of bacteria known to regulate their toxin production with a general cell-density cue. Ahead of such experiments, our modelling work has provided more insight into the mechanisms underlying the evolution of complex regulation systems in microbial populations.

## Methods

The general set-up of the model is discussed in the Model section. Below, we provide details on the implementation and analysis. The model was implemented in C. Code is available from the corresponding author upon request.

### Spatially structured individual-based model

We developed a spatially explicit individual-based model of bacteria evolving their production of an anticompetitor toxin, resistance, and response to a cell-density cue. Bacteria in the model are characterised by a genotype of four characteristics: a toxin production gene, a resistance gene, a toxin production rate *π*_T_ and a cue response threshold *θ* (Fig 1A). The bacteria live on a square *N* × *N* lattice (*N* = 512 for all simulations in this paper) with periodic boundary conditions. Each lattice site can contain at most one bacterium.

#### Concentration profiles of the cell-density cue and the toxin

The production, degradation and diffusion of the cell-density cue and the toxin are described with partial differential equations. Let $c_{k}\left(\vec{x},t\right)$ be the concentration of secreted product *k* at location $\vec{x}$ at time *t*, where the index *k* can be replaced by “cue” or “toxin”. Then

$$\begin{array}{c} \frac{\partial c_{k}\left(\vec{x},t\right)}{\partial t}=\underset{\text{production}}{\underset{︸}{P_{k}\left(\vec{x},t\right)}}-\underset{\text{degradation}}{\underset{︸}{d_{k}c_{k}\left(\vec{x},t\right)}}+\underset{\text{diffusion}}{\underset{︸}{D_{k}\nabla^{2}c_{k}\left(\vec{x},t\right)}}, \end{array}$$ (1)

where *d*_*k*_ is the degradation rate, *D*_*k*_ the diffusion rate, and $P_{k}\left(\vec{x},t\right)$ the production rate at location $\vec{x}$. For simplicity, we assume that the cue molecule is produced by all bacteria at a fixed rate *p*_cue_: $P_{\text{cue}}\left(\vec{x},t\right)$ equals *p*_cue_ if the lattice site at location $\vec{x}$ is occupied, and is 0 otherwise (similar to previous implementations of density cue dynamics [26, 47]). For the toxin, $P_{\text{tox}}\left(\vec{x},t\right)$ equals $\pi_{T}\left(\vec{x}\right)$, *i.e*., the toxin production rate of the bacterium at location $\vec{x}$, if the lattice site is occupied by a bacterium expressing its toxin production gene, and is 0 otherwise.

Each simulation time step, the quasi-steady-state concentration profile $c_{k}\left(\vec{x}\right)$ of molecule *k* is found by equating the right-hand side of Eq 1 to zero and solving for *c*_*k*_. Taking the 2D finite-difference approximation of Eq 1 and dropping the index *k* for brevity, this yields

$$\begin{array}{c} P_{i,j}-dc_{i,j}-4Dc_{i,j}+D\left(c_{i-1,j}+c_{i+1,j}+c_{i,j-1}+c_{i,j+1}\right)=0, \end{array}$$ (2)

for each position (*i*, *j*) on the simulation lattice. Here, *P* is the production matrix containing the production rates of the molecule at each position, *d* is the degradation rate and *D* the diffusion rate. Since we consider periodic boundary conditions, all indices in Eq 2 should be read modulo *N*.

Eq 2 is a linear system of *N* × *N* equations. To solve it efficiently, we make use of Fourier transformations. Because we assume a square simulation lattice and periodic boundary conditions, *c*_*i*,*j*_ is a discrete periodic function with period *N* in both indices. Let *G* be the degradation-diffusion kernel at (1, 1):

$$\begin{array}{c} G≔(\begin{array}{ccccc} -4D-d & D & 0 & \cdots & D\\ D & 0 & 0 & \cdots & 0\\ 0 & 0 & 0 & \cdots & 0\\ \vdots & \vdots & \vdots & \ddots & \vdots \\ D & 0 & 0 & \cdots & 0 \end{array}), \end{array}$$ (3)

such that *G*_*i*,*j*_ describes the diffusion and degradation effects of the molecule concentration in lattice site (*i*, *j*) on the concentration in lattice site (1, 1). The *N* × *N* equations defined by Eq 2 can then be rewritten as

$$\begin{array}{c} P+\left(c{*}_{N}G\right)=0,\;\text{or}\;c{*}_{N}G=-P, \end{array}$$ (4)

where c*_N_ *G* is the circular discrete convolution of matrices *c* and *G*. By the circular convolution theorem, c*_N_ *G* is equal to the inverse Fourier transform of the element-wise product of the individual Fourier transforms of *c* and *G*, *i.e*. we can solve F(c) from

$$\begin{array}{c} F(c)\cdot F(G)=F(P), \end{array}$$ (5)

and then compute the inverse Fourier transform to get the concentration matrix *c*. Since the degradation and diffusion rates of the molecules are constant over the simulation, so is *G*. Hence, F(G) needs to be calculated only once per simulation, and at each simulation time step we are left with finding the Fourier transform F(P) of the production matrix, dividing the result element-wise by F(G), and then using the inverse Fourier transform to find *c*. Fourier transformations were performed using the fftw3-library [62]. Since algorithms for Fourier transformations are highly efficient [62], Eq 5 allows us to rapidly find the quasi-steady-state concentration profiles even for large simulation lattices.

#### Population dynamics of bacteria

Every time step, the phenotype of bacteria (toxin production *ϕ*_T_, resistance *ϕ*_R_, and cue response *ϕ*_C_) is determined based on their genotype and the current local cue concentration (Fig 1A). If a gene is regulated, it is expressed only if the cue concentration exceeds the cell’s response threshold *θ*.

The probability of cell death per time step has a basal value *δ* and increases linearly with the local toxin concentration $c_{\text{tox}}\left(\vec{x}\right)$, unless the bacterium is resistant:

$$\begin{array}{c} P\left(\text{death}\;\text{of}\;\text{cell}\;j|\vec{x}\right)=\delta +\left(1-\phi_{R_{j}}\right)\delta_{\text{tox}}c_{\text{tox}}\left(\vec{x}\right). \end{array}$$ (6)

The slope *δ*_tox_ describes the toxicity of the toxin. When bacteria die, they leave behind an empty lattice site.

Empty sites can be repopulated by reproduction of bacteria on the eight neighbouring lattice sites. For each empty lattice site, the probability that it becomes occupied by reproduction of any of its neighbouring bacteria in the current time step is

$$\begin{array}{c} P\left(\text{reproduction}|\vec{x}\right)=1-e^{-\gamma \frac{1}{8}\sum_{i\in \text{neighbours}}R_{i}}, \end{array}$$ (7)

where *γ* is a scaling factor determining the maximal reproduction rate per time step and *R*_*i*_ is the reproductive fitness of the *i*-th neighbour (further specified below). If reproduction occurs, neighbour *j* is selected as the parent with probability

$$\begin{array}{c} P\left(\text{reproduction}\;\text{of}\;\text{cell}\;j|\vec{x}\right)=\frac{R_{j}}{\sum_{i\in \text{neighbours}}R_{i}}. \end{array}$$ (8)

Eqs 7 and 8 ensure that (i) the overall probability of reproduction increases with the total reproductive fitness of the eight neighbours but never exceeds 1, and (ii) the probability of reproduction of a specific cell is determined by its reproductive fitness relative to its local competitors.

Toxin production, resistance, and the ability to respond to the density cue bear metabolic costs. The metabolic costs of cell *j*’s phenotype are linearly incorporated in its reproductive fitness as

$$\begin{array}{c} R_{j}=\text{max}[0,(1-\;\phi_{T_{j}}\underset{\text{toxin}\;\text{production}\;\text{cost}}{\underset{︸}{\left(C_{T_{0}}+b_{T}\pi_{T_{j}}\right)}}\;-\;\phi_{R_{j}}\underset{\text{resistance}\;\text{cost}}{\underset{︸}{C_{R}}}\;-\;\phi_{C_{j}}\underset{\text{response}\;\text{cost}}{\underset{︸}{C_{C}}})]. \end{array}$$ (9)

The costs for resistance (*C*_R_) and the ability to respond to the cue (*C*_C_) are given by single parameters. The cost for toxin production linearly depends on the cell’s toxin production rate *π*_T_, with offset $C_{T_{0}}$ and slope *b*_T_. Note that we do not include a cost for cue production. Because all cells produce the density cue (at the same rate), such a cost would be the same for all phenotypes, and hence would not influence the competition between cells of different phenotypes.

#### Mutations

When a bacterium reproduces, the daughter cell generally inherits the genotype of its parent. With small probability, however, mutations are introduced. First, mutations can alter the toxin and resistance gene. If a mutation generates a cell that produces toxin while not simultaneously expressing resistance, this cell is considered nonviable and discarded from the simulation. Second, mutations in the response threshold value *θ* are introduced with a fixed probability *μ*. If the parent cell has a regulating genotype, the mutated response threshold is generally drawn from a uniform distribution on the interval [*θ*_parent_ − *σ*_*μ*_; *θ*_parent_ + *σ*_*μ*_] (if the new threshold value is below 0, it is set to 0). However, to ensure that the genotype space is sufficiently accessible, with probability *p*_largemut_ = 10^−3^ the new response threshold is chosen randomly between 0 and 1. Response threshold values of regulating daughter cells with a non-regulating parent are also uniformly sampled from [0, 1]. Third, mutations in the toxin production rate *π*_T_ are introduced in the same way as response threshold mutations, with the same rate *μ*, step size *σ*_*μ*_, and probability of not inheriting the parental value *p*_largemut_.

The number of ways a functional sequence can be removed or destroyed by small-scale mutations (substitutions, short indels) usually greatly exceeds the number of mutations that can create such a functional sequence, simply because most sequences do not perform the desired function. For larger scale mutations, gene loss is thought to be the major driver of evolution of many prokaryotic lineages over relatively short evolutionary times (*e.g*., within genera), occurring several times more frequently than *de novo* gene discovery and gene gain through horizontal gene transfer [63, 64]. We therefore consider gain-of-function mutations (Off → On, Off → Reg, and On → Reg) to be less likely than loss-of-function mutations (On → Off, Reg → Off, and Reg → On).

### Parameter sweep

#### Parameter reduction and parameter values

Altogether, the model has 18 parameters. Brute-force sampling an 18-dimensional parameter space in an exploratory parameter sweep is computationally infeasible. Fortunately, the parameter space can be reduced by identifying lumped parameters (Table 1, see S1 Text for derivations). The 13 parameters remaining after the parameter reduction include the 5 mutation parameters and the delay between expression of resistance and toxin in (Reg, Reg)-cells *τ*_delay_. Over many test runs we observed that these parameters only marginally affect the simulation results, as long as mutations happen reasonably frequently. The mutation parameters and *τ*_delay_ were therefore fixed at default values (Table 1). To further reduce the number of parameters included in our parameter sweep, we finally noted that the offset $C_{T_{0}}$ defines a minimal cost of toxin production and should hence be > 0 to make sure toxin production is never “free”, but should also not be too large because else toxin production can never evolve. Because ${\hat{b}}_{T}$ is also a measure for the cost of toxin production, we chose to keep $C_{T_{0}}=0.01$ constant, and only vary ${\hat{b}}_{T}$ in the parameter sweep along with the five other remaining parameters (Table 1).

To capture the different potential evolutionary outcomes for varying bacterial species and environments, we varied our parameters over relatively broad ranges in a parameter sweep (Table 1). Since we aimed to investigate regulation by local cell density, the characteristic length scale *L*_cue_ of the density cue was chosen between 2 and 20 lattice sites. To set reasonable values for the toxin length scale *L*_tox_, we noted that in many KRS-models toxin-dependent killing is limited to direct neighbours in space [7, 11, 29, 65]. Experiments show, however, that the inhibition range of toxins is generally much larger, and can span several tens or even hundreds of *μ*m [6, 56]. For colicins, a length scale of 100—175 *μ*m has been reported [44], which corresponds in size to at least 50 bacteria. We therefore chose the range of *L*_tox_ twice as broad as the range for *L*_cue_ (Table 1). KRS-systems in general, and regulation of toxin production in particular, evolved more readily for small values of *L*_tox_ (see Fig 3), so an even wider range would not yield more insight into the model.

To determine bounds on *δ*/*γ*, note that this lumped parameter is equal to *R*_0_^−1^ of the bacteria, where *R*_0_ is the maximal expected number of offspring of a sensitive cell (*i.e*. when it is completely surrounded by empty space). Any population with *R*_0_ < 1 is nonviable. Note, furthermore, that for a sensitive population at carrying capacity the cell density is equal to 1 − *R*_0_^−1^. Since toxin production is most likely to evolve in high-density environments where competition for empty space is strong, the range of *δ*/*γ* was chosen such that the density of cells at carrying capacity is at least 50%.

For the three cost parameters, a lower bound of 0.01 was chosen to avoid the occurrence of “free” phenotypes that are not selected for. Metabolic costs for toxin production and resistance have been shown to exist for various toxins, although the extent of these costs may differ between toxins and bacterial species [6–8, 44, 66]. To capture these different scenarios, we vary the toxin production cost and resistance cost parameters between the lower bound of 0.01 and relatively high values. For the resistance cost, we go up to a cost of 25% of the reproduction rate, while for the toxin production we set an upper bound on ${\hat{b}}_{T}$ such that the reproduction rate of producing cells would be 0 if its production rate *π*_T_ = 1. Since we expect regulation to evolve only if the costs of regulation are lower than the costs of the regulated behaviours (toxin production and resistance), we consider a narrower range for the cue response cost *C*_C_ (Table 1).

#### Simulations and analysis

To sample the parameter space, 2000 simulations were performed for random combinations of the six lumped parameters. Parameter values were independently sampled from uniform distributions with a wide, parameter-specific range (Table 1). Simulations were initialised with a randomly selected 10% of lattice sites occupied by cells with random genotypes (response threshold values and the toxin production rate values were randomly chosen between 0 and 1), and were run for 400, 000 time steps (generally sufficient to reach evolutionary steady state). The mean fraction of cells with each possible geno-/phenotype combination was calculated over the last 50, 000 simulation time steps. Based on these genotype and phenotype abundances the simulations were classified using a decision tree with several steps (S1 Fig). In the first step, simulations were assessed on the abundance of phenotypes (S: sensitives, R: resistants, or K: killers), and a phenotype was called “fixed” if it was present at > 98% abundance. Only fixation of sensitives was observed; resistance or toxin production never fixed. If cells of all three possible phenotypes (K, R and S) occurred at appreciable abundance (defined as > 2% of the population), the simulation was classified as “KRS”. Simulations that did not yield fixation of a single phenotype or KRS-dynamics were classified as “other”. In the second step, simulations within the KRS-class were considered to show “potential regulation” if at least one regulating genotype was present at > 2% abundance. In the final third step, these “potential regulation” cases were only classified as “true regulation” if at least 10% of the cells of the regulating genotype were in the inactive phenotype (regulated gene not expressed), and at least 10% were in the active phenotype. Altogether, this classification assigned all simulations to one of four evolutionary outcomes: (1) Sensitives fix, (2) KRS-dynamics, no regulation evolved, (3) KRS-dynamics, regulation evolved, and (4) other (S1 Fig).

### Invasion speeds

To understand why the regulating killer cells (genotype (Reg, On)) can outcompete constitutive killer cells (genotype (On, On)) under certain conditions, we compared invasion dynamics of these two killer types. To allow for a fair comparison, we first evolved constitutive killers under conditions that would usually favour regulation by removing the possibility of regulation from the model (S3A Fig, evolved under the same parameter conditions as Fig 4). Over three replicate simulations, constitutive killers under these conditions evolved a mean toxin production rate of *π*_T_ = 0.13. Over the ten replicate simulations of evolving (Reg, On)-killers, these cells evolved a mean toxin production rate of *π*_T_ = 0.8 and a mean response threshold of *θ* = 0.875 (S2 Fig). We therefore constructed two “average evolved killer strains”, a constitutive killer with genotype (On, On) and *π*_T_ = 0.13, and a regulating killer with genotype (Reg, On), *π*_T_ = 0.8 and *θ* = 0.875, and compared the invasion dynamics of these two constructed killer strains.

To characterise the invasion into a sensitive population, a 20-cell-wide strip of one of the two killer strains was placed on a lattice that was otherwise filled with a sensitive population at carrying capacity (S3B Fig). Population dynamics were then simulated and the decline of the number of sensitives over time was followed (S3B Fig). The invasion speed was calculated as

$$\begin{array}{c} v_{{\text{inv}}_{S}}=\frac{-\beta_{S\;\text{on}\;t}}{K_{S}N}\frac{\text{lattice}\;\text{sites}}{\text{time}}, \end{array}$$ (10)

where *β*_*S* on *t*_ is the linear regression coefficient of the number of sensitive cells on time, *N* is the number of rows of the simulation lattice and *K*_S_ = (1 − *δ*/*γ*) is the density of sensitive cells at carrying capacity. Similarly, the invasion speed of resistant cells (genotype (Off, On)) into a population of (Reg, On)-cells and (On, On)-cells was measured by placing a 20-cell-wide strip of resistant cells on a lattice otherwise filled with (Reg, On)-cells or (On, On)-cells at carrying capacity and calculating

$$\begin{array}{c} v_{{\text{inv}}_{R}}=\frac{\beta_{R\;\text{on}\;t}}{K_{R}N}\frac{\text{lattice}\;\text{sites}}{\text{time}}, \end{array}$$ (11)

where *β*_*R* on *t*_ is the linear regression coefficient of the number of resistant cells on time and *K*_R_ = (1 − *δ*/(*γ*(1 − *c*_R_)) is the density of sensitive cells at carrying capacity. (The invasion speeds we measure here serve as a tool to quantify the difference between the two killer strains and thus better understand the evolutionary outcome of the simulations. For a more formal analysis of the effect of toxin production and quorum sensing on invasion speeds, see [67]).

Note that to calculate $v_{{\text{inv}}_{S}}$ the decline of the number of sensitives is used, while in the calculation of $v_{{\text{inv}}_{R}}$ the increase in the number of resistant cells is considered. This choice was made because the characteristics of the sensitive strain and the resistant strain are the same in both invasion experiments, while the two killer strains differ. For each invasion experiment, 10 replicate runs were performed.

### Serial transfers

Under the serial transfer regime, simulations were again initialised with cells with random genotypes placed at a random 10% of lattice sites. Population dynamics were simulated as before, except that the simulations were periodically paused and a transfer was performed. At each transfer, a random sample of the population at the end of the growth cycle was taken as founder cells for the new population. These founder cells were then randomly placed on an otherwise empty simulation lattice, and the simulation of the population dynamics was resumed until the next transfer. Unless otherwise noted, transfers were performed every 500 simulation time steps, and each new cycle was seeded with 1000 founder cells. Simulations were continued for 800 (parameter sweep) or 1200 (example runs) transfer cycles. Evolutionary steady state was generally reached well before the end of the simulation.

## Supporting information

S1 Text Parameter reduction. (PDF)

S2 Text Analytical approximation of the cue concentration profile in a single growing colony. (PDF)

S1 Video KRS-dynamics with a regulating killer type. Dynamics of an example simulation that yielded KRS-dynamics with regulating killers. Parameter settings as in Fig 4. The concentration of the density cue (left panel), the spatial distribution of cells (central panel), and the concentration of the toxin (right) are shown. Cells are colour-coded for their genotype and phenotype, see Fig 5 for legend. A dynamic steady state is reached in which three genotypes coexist: sensitives (genotype (Off, Off), blue), resistants (genotype (Off, On), white) and cells that regulate their toxin production (genotype (Reg, On), dark orange when toxin production phenotype *ϕ*_T_ = 1, light orange when *ϕ*_T_ = 0). These genotypes follow KRS-dynamics, with the (Reg, On)-cells in the role of killers. Within patches of the (Reg, On) genotype, cells frequently switch between a resistant and toxin producing phenotype.
(MP4)

S2 Video Model dynamics under a serial-transfer regime. Parameter settings as in Fig 5, panels and legend as in S1 Video. Because dynamics under transfers are much faster than in the fixed environment, this video runs 20 times slower than S1 Video. After a transfer, colonies rapidly grow into the empty space. Cells of the three most abundant genotypes, (Off, Off), (Off, Reg) and (Reg, Reg), all initially have a sensitive phenotype. In the interior of growing colonies and wherever colonies meet, the concentration of the density cue increases and regulating cells switch to a resistant and/or toxin producing phenotype.
(MP4)

S3 Video Growth dynamics of and interactions between colonies of the (Off, Off)-, (Off, Reg)- and (Reg, Reg)-cells that evolved under serial transfers. To better illustrate the colony dynamics under serial transfers, we seeded three colonies at equal distances, each with one of the three genotypes that are found in the evolved population (see Fig 5 and S7 Fig): (i) the sensitive genotype ((Off, Off)-cells), (ii) the regulating resistant genotype ((Off, Reg)-cells, *θ* = 0.67), and (iii) the regulating killer genotype ((Reg, Reg)-cells, *π*_T_ = 1, *θ* = 0.67). Bacteria are coloured based on their genotype and phenotype as in Fig 5. Cells in all colonies initially express a sensitive phenotype. As the colonies expand, the concentration of the density cue inside the colonies increases. After some time, regulating cells in the colonies’ interior therefore switch to a resistant phenotype and, in the case of (Reg, Reg)-cells, subsequently to a toxin producing phenotype. Where two expanding colonies collide, the local cell density is also high so that regulating cells express their resistant or toxin producing phenotype. At the interfaces between the colonies, KRS-dynamics emerge: the regulating killer colony slowly invades the sensitive colony, the sensitive colony slowly invades the regulating resistant colony, and the regulating resistant colony slowly invades the regulating killer colony.
(MP4)

S1 Fig The evolutionary outcome of runs in the parameter sweep was classified based on genotype and phenotype abundance. For 2000 different parameter combinations a simulation was run for 400000 time steps, and for each simulation the mean abundance of genotypes and phenotypes in the last 50000 time steps was calculated. Based on these abundance distributions, simulations were classified as showing one of four possible evolutionary outcomes: (i) the sensitive genotype (Off, Off) fixed, (ii) KRS-dynamics arose, no regulation evolved, (iii) KRS-dynamics arose, regulation evolved, and (iv) “other”. This classification was performed in several steps: (1) considering the abundance of different phenotypes in the population (sensitive / resistant / toxin producing), (2) asking if any regulating genotype was present at appreciable abundance (≥ 2% of the population), and (3) asking if such a regulating genotype expressed both of its potential phenotypes (both phenotypes expressed by at least 10% of the regulating cells). This final step ensures that cells identified as regulators indeed switch between phenotypes.
(PDF)

S2 Fig Evolution of regulation in a fixed habitat is highly reproducible. Results of ten independent replicates of the simulation shown in Fig 4. Simulations were run for 400000 time steps, and the genotype distribution was calculated from the mean abundance of genotypes in the last 50000 simulation time steps. In all runs, a KRS-system evolved with regulating (Reg, On)-killer cells, and the genotype distribution at steady state is very consistent over replicates. The evolved toxin production rate did vary somewhat over replicates, but 0.5 < *π*_T_ < 1.0 in all simulations (middle panel). The distribution of response threshold values *θ* in the (Reg, On)-cells at the end of the simulation is highly consistent over replicates (bottom panel).
(PDF)

S3 Fig Regulation provides the evolved (Reg, On)-cells with an advantage over constitutive killers both when invading sensitives and in the competition with resistant cells. (A) To allow for a fair comparison with the evolved (Reg, On)-cells, constitutive killer cells (genotype (On, On)) were evolved under the same parameter conditions as Fig 4. The example shown here is representative of three replicate runs. (B) Invasion experiments were initialised by placing a 20-cell wide strip of the invading strain in a simulation lattice otherwise filled with the to-be-invaded strain at carrying capacity. The illustration shows the invasion of the (On, On)-strain and the (Reg, On)-strain in a sensitive population; similar experiments were performed for the invasion of a resistant strain in an (On, On)- or (Reg, On)-population. Invasion speed *v*_inv_ was measured as the decline in the number of sensitives over time, or as the increase of the number of resistant cells over time. (C) Invasion speed of the (Reg, On)-strain into sensitives is higher than the invasion speed of the (On, On)-strain, while invasion speed of the resistant strain is lower in a (Reg, On)-population than in an (On, On)-population. Mean invasion speed ±2 SEM is shown for 10 replicate invasion experiments per combination of invading and invaded strain.
(PDF)

S4 Fig Regulation allows the evolved (Reg, On)-cells to produce toxin only when few of their neighbouring sites are empty. The number of empty neighbouring lattice sites was counted for evolved (Reg, On)-cells at steady state (end of simulation in Fig 4). (A) The concentration of the density cue is highest when cells have no empty neighbours, and decreases with the number of empty neighbours. The mean evolved response threshold value (*θ* = 0.875) is indicated by a dotted line. Of the cells with no empty neighbours, over half sensed a cue concentration > *θ*, whereas of the cells with 4 or more empty neighbours, none did. (B) The proportion of cells currently producing toxin as a function of their number of empty neighbours. Around 50% of cells without any empty neighbours produce toxin, while (almost) no cells produce toxin when 3 or more of their neighbouring sites are empty.
(PDF)

S5 Fig Regulation evolves only if phenotypic adaptation is sufficiently fast. The simulations were repeated for cells that cannot instantaneously adjust their phenotype to the sensed cue concentration, but rather have a lag time between sensing a change in cue concentration and expressing the corresponding phenotype. For each value of this lag time, 5 replicate runs were performed and the genotype distribution was calculated from the mean abundance of genotypes in the last 50000 simulation time steps (evolutionary steady state). For a relatively short lag (5 time steps, which is equivalent to 50% of the minimal bacterial doubling time), regulation still evolved in 3 out of 5 replicates. For longer lag times (≥ 10 time steps), no regulation was found.
(PDF)

S6 Fig Parameter sweep results under a serial-transfer regime. Simulations were run for the same 2000 parameter settings as used for S1 Fig and Fig 3, with the exception that serial transfers were performed once every 500 time steps, reseeding the new population with 1000 founder cells. (A) Table of simulation outcomes, classified as indicated in S1 Fig. Under the serial-transfer regime the sensitive (Off, Off)-phenotype fixes under more parameter conditions than when the simulation is performed in a constant environment, but regulation also evolves more frequently. (B) Parameter conditions in simulations with different outcomes. Significance is shown for 2-sided *t*-tests with Bonferroni correction for multiple testing: ***: *p* < 10^−10^, **: *p* < 10^−3^, *: *p* < 0.05, n.s.: not significant. Toxin production of any type (regulated or non-regulated) is found only when the natural death rate of bacteria is low and phenotypic costs, especially of toxin production, are also low. Among the simulations that resulted in KRS-dynamics, simulations in which regulation evolved have higher toxin and resistance cost and lower response cost than simulations that did not yield regulation. These conditions are similar to the conditions for regulation in the fixed habitat (*c.f*., Fig 3).
(PDF)

S7 Fig Evolution of regulation under a serial-transfer regime is highly reproducible. Independent replicate runs of the simulation shown in Fig 5. The top panel shows the genotype abundance profile, which was calculated as the mean proportion of genotypes in the population over the last 50000 time steps of the simulation. The middle and bottom panel show the mean evolved toxin production rate of (Reg, Reg)-cells and the distribution of evolved response threshold values in (Reg, Reg)- and (Off, Reg)-cells at the end of the simulation (Time = 600000). Some quantitative variation exists between replicates, especially in the evolved toxin production rate. However, in all replicates (Off, Off)-, (Off, Reg)- and (Reg, Reg)-cells are selected with similar response threshold values.
(PDF)

S8 Fig Evolution of regulation under serial transfers is robust to variations in the time between transfers and the number of founder cells. Simulations were performed with parameter conditions as in Fig 5, with the exception of the time between transfers or number of founder cells, which were varied. For each parameter setting, five independent replicate runs were performed. The mean proportion of genotypes over the last 50000 time steps was calculated in the pooled population of all cells in these five replicates. (A) Results of varying the time between transfers. When transfers are very frequent sensitive cells dominate the population, while if transfers are very infrequent non-regulating killer, resistant and sensitive cells are found. However, under a wide range of intermediate transfer intervals regulation readily evolves. (B) Results of varying the number of founder cells. When the population is seeded with very few cells after a transfer, only sensitive cells are selected, while when the number of founder cells is very large a non-regulating KRS-system arises. Again, regulation does evolve for a wide range of intermediate numbers of founder cells.
(PDF)

S9 Fig Regulation also evolves if transfers happen stochastically. Instead of a fixed time interval between transfers, the length of each transfer cycle was drawn independently from an geometric distribution with mean *τ*_transfer_ = 500. This way, the mean length of a cycle was kept constant, but transfers now happened at a fixed probability per time step. Other settings were the same as in Fig 5. (A) Population dynamics over a relatively short time interval, illustrating the irregular transfers. (B) Simulation results for five independent replicate simulations. In all five replicates regulation evolved. The results are very similar to the case with regular transfer (Fig 5 and S7 Fig).
(PDF)

S10 Fig Regulation under serial transfers also evolves when there is no delay between expression of resistance and toxin production. Evolved genotype distribution for varying values of the delay between expression of resistance and toxin production in (Reg, Reg)-cells, *τ*_delay_ (mean outcome of 5 replicate runs per *τ*_delay_ value). Regulation of toxin production and resistance (genotype (Reg, Reg)) still evolves when there is no such delay (*τ*_delay_ = 0 time steps), and when the delay is up to three times higher than the default value (*τ*_delay_ = 150 time steps).
(PDF)

S11 Fig Regulation still evolves under serial transfers when there is substantial lag between the change in cue concentration and the corresponding change in phenotype in regulating cells. A lag time between cue sensing and phenotype adjustment was implemented as in S5 Fig, and simulations were run for varying values of this lag (5 replicate simulations per lag time value). Under serial transfers, the evolution of regulation is robust to relatively long lag times: regulation still evolved for lag times up to 30 time steps, or 3 bacterial doubling times.
(PDF)

S12 Fig Under serial transfers, regulation is highly robust to noise in the cue concentration. Simulations were run with the same settings as Fig 5, but at each time point at each lattice site a Gaussian noise term (mean *μ*_noise_ = 0, standard deviation *σ*_noise_ = 0.1) was added to the local concentration of the density cue. (A) Simulation results of a single, representative run. (B) Summarised results of five replicate runs. Regulation evolved in all five replicate runs. The noise term substantially increased the variation in cue concentrations sensed by cells (grey distribution in right panel of A; note the larger range on the *y*-axis (*c.f*., Fig 6)). The evolved response threshold values are however very similar to the values found in the absence of noise (red distribution in right panel of A, compare to Fig 6).
(PDF)
