# Supplementary material for: Toxin production spontaneously becomes regulated by local cell density in evolving bacterial populations
Source: PLoS Comput Biol. 2019 Aug 30;15(8):e1007333. doi: 10.1371/journal.pcbi.1007333 (PMC6742444; doi:10.1371/journal.pcbi.1007333)
Supplement: S1 Text — (PDF) [file pcbi.1007333.s001.pdf]

Supporting Text S1

Toxin production spontaneously becomes regulated by local cell density in evolving bacterial populations

Hilje M. Doekes[1,*], Rob J. de Boer[1], Rutger Hermsen[1]
1 Theoretical Biology, Department of Biology, Utrecht University, Padualaan 8, 3584 CH Utrecht, The Netherlands.
* hiljedoekes@gmail.com

Parameter reduction

To efficiently sample the parameter space in a parameter sweep, the number of parameters was reduced by convenient scaling of the variables. We here consider the cue and toxin concentration profiles and the population dynamics of the bacteria, but do not include mutations and the delay between expression of toxin and resistance, τ_{delay} (See Model and Methods sections of the main text).
In the simulations, time progresses in discrete steps, and probabilities for reproduction and death of bacteria are calculated for each time step (Methods, Eq 6-9). Because we ensure that these probabilities are always small, the simulated discrete-time process approximates a continuous-time Poisson process with (time-dependent) reproduction and death *rates*. This association with a continuous-time process is necessary to allow the time variable to be scaled continuously.
In particular, let Δt be the simulation time step. Then the death rate d_k of cell k is given by

$$d_k \approx \frac{\mathbb{P}(\text{death of cell } k \text{ in simulation time step})}{\Delta t} = \frac{\delta + (1-\phi_{\text{R}_k})\delta_{\text{tox}} c_{\text{tox}}(\vec{x}_k)}{\Delta t}. \tag{S1.1}$$

For the reproduction dynamics (Methods, Eq 7-9), the scaling factor γ is always chosen $\ll 1$, which ensures that the probability of reproduction per time step is small. Then, the probability of reproduction into an empty lattice site is approximately linear in the sum of the reproductive fitnesses R_i of the neighbouring bacteria:

$$\mathbb{P}(\text{reproduction}|\vec{x}) \approx \frac{\gamma}{8} \sum_{i \in \text{neighbours}} R_i,$$

and the probability that a specific neighbouring bacterium k reproduces into the empty site is $\frac{1}{8}\gamma R_k$. Since a bacterium can reproduce into any empty neighbouring site, the expected number of offspring of a given cell k in a time step is $f_{\text{empty}}\gamma R_k$, where f_{empty} is the fraction of its eight neighbouring lattice sites that is empty. Conventionally defining a cell's reproduction rate r_k as the rate at which a cell reproduces when surrounded by empty space, we then find

$$r_k \approx \frac{\gamma R_k}{\Delta t} = \frac{\max\Big[0, \gamma\big(1 - \phi_{\text{T}_k}(C_{\text{T}_0} + b_{\text{T}}\pi_{\text{T}_k}) - \phi_{\text{R}_k} C_{\text{R}} - \phi_{\text{C}_k} C_{\text{C}}\big)\Big]}{\Delta t}. \tag{S1.2}$$

The concentration profiles of the density cue and toxin are found from

$$P_{i,j} - d_{\text{cue}} c_{\text{cue}} + D_{\text{cue}} \nabla^2 c_{\text{cue}} = 0, \tag{S1.3}$$
$$\text{where } P_{i,j} = \begin{cases} p_{\text{cue}} & \text{if there is a cell at position } (i,j), \\ 0 & \text{if not,} \end{cases}$$

and

$$\pi_{T_{i,j}} - d_{\text{tox}} c_{\text{tox}} + D_{\text{tox}} \nabla^2 c_{\text{tox}} = 0, \tag{S1.4}$$
$$\text{where } \pi_{T_{i,j}} = \begin{cases} \pi_T & \text{of the cell at position}(i,j) \text{ if that cell produces toxin,} \\ 0 & \text{otherwise.} \end{cases}$$

Taken together, the simulation dynamics are hence captured by four equations: Eq S1.1 – S1.4.
To reduce the number of parameters in Eq S1.1 – S1.4, we introduce the scaled variables

$$\tau = \frac{\gamma}{\Delta t} t, \tag{S1.5}$$

$$\hat{c}_{\text{cue}} = \frac{d_{\text{cue}}}{p_{\text{cue}}} c_{\text{cue}}, \tag{S1.6}$$

$$\hat{c}_{\text{tox}} = \frac{\delta_{\text{tox}}}{\gamma} c_{\text{tox}}, \tag{S1.7}$$

$$\hat{\pi}_T = \frac{\delta_{\text{tox}}}{\gamma d_{\text{tox}}} \pi_T. \tag{S1.8}$$

In terms of these variables, the equations for the cue and toxin concentration reduce to

$$\hat{P}_{i,j} - \hat{c}_{\text{cue}} + \tfrac{1}{4} L_{\text{cue}}^2 \nabla^2 \hat{c}_{\text{cue}} = 0, \tag{S1.9}$$

$$\text{where } \hat{P}_{i,j} = \begin{cases} 1 & \text{if there is a cell at position } (i,j), \\ 0 & \text{if not,} \end{cases}$$

and

$$\hat{\pi}_{T_{i,j}} - \hat{c}_{\text{tox}} + \tfrac{1}{4} L_{\text{tox}}^2 \nabla^2 \hat{c}_{\text{tox}} = 0, \tag{S1.10}$$

$$\text{where } \hat{\pi}_{T_{i,j}} = \begin{cases} \hat{\pi}_T & \text{of the cell at position}(i,j) \text{ if that cell produces toxin,} \\ 0 & \quad\text{otherwise,} \end{cases}$$

with only one lumped parameter per equation:

$$L_{\text{cue}} = 2\sqrt{\frac{D_{\text{cue}}}{d_{\text{cue}}}},$$

$$L_{\text{tox}} = 2\sqrt{\frac{D_{\text{tox}}}{d_{\text{tox}}}}.$$

The parameters L_{cue} and L_{tox} represent the characteristic length scales of the concentration profiles; they are equal to the mean square displacement of the diffusing cue or toxin molecules before they are degraded. The death and reproduction rates have the dimension of 1/time. Therefore, after scaling time according to Eq S1.5 the scaled death rate reads

$$\hat{d}_k = \frac{\Delta t}{\gamma} d_k = \tfrac{\delta}{\gamma} + (1 - \phi_{\text{R}_k}) \hat{c}_{\text{tox}}(\vec{x}_k), \tag{S1.11}$$

while the reproduction rate reduces to

$$\hat{r}_k = \frac{\Delta t}{\gamma} r_k = \max\Big[0, 1 - \phi_{\text{T}_k}(C_{\text{T}_0} + \hat{b}_{\text{T}} \hat{\pi}_{\text{T}_k}) - \phi_{\text{R}_k} C_{\text{R}} - \phi_{\text{C}_k} C_{\text{C}}\Big], \tag{S1.12}$$

with lumped parameter

$$\hat{b}_{\text{T}} = \frac{\gamma d_{\text{tox}} b_{\text{T}}}{\delta_{\text{tox}}}.$$

After scaling, seven parameters are left in the description of the system (Eq S1.9 – S1.12): the characteristic length scales of the concentration profiles L_{cue} and L_{toxt}, the ratio $\frac{\delta}{\gamma}$, which is equal to R_0^{-1} with R_0 the maximum number of expected offspring per bacterial life time, the scaled slope of the toxin production cost function $\hat{b}_{\text{T}}$ and its offset C_{T_0}, the cost of resistance C_{R}, and the cost of responding to the cue C_{C}.
