# Supplementary material for: Toxin production spontaneously becomes regulated by local cell density in evolving bacterial populations
Source: PLoS Comput Biol. 2019 Aug 30;15(8):e1007333. doi: 10.1371/journal.pcbi.1007333 (PMC6742444; doi:10.1371/journal.pcbi.1007333)
Supplement: S2 Text — (PDF) [file pcbi.1007333.s002.pdf]

Supporting Text S2

Toxin production spontaneously becomes regulated by local cell density in evolving bacterial populations

Hilje M. Doekes[1,*], Rob J. de Boer[1], Rutger Hermsen[1]
1 Theoretical Biology, Department of Biology, Utrecht University, Padualaan 8, 3584 CH Utrecht, The Netherlands.
* hiljedoekes@gmail.com

Analytical approximation of the cue concentration in a single growing colony

Here, we present an analytical approximation for the cue concentration profile of a single colony, which we use to derive a theoretical maximum for the cue concentration experienced by cells at the colony edge. Analytical solutions were found using the `DSolve`, `Solve` and `Limit` functions of Wolfram Mathematica 11.0.

Consider a growing, genotypically homogeneous colony of model bacteria (Fig S2.1A). To find the cue concentration profile of this colony, we should solve

$$\frac{\partial c_{\text{cue}}(\vec{x},t)}{\partial t} = P_{\text{cue}}(\vec{x},t) - d_{\text{cue}}c_{\text{cue}}(\vec{x},t) + D_{\text{cue}}\nabla^2 c_{\text{cue}}(\vec{x},t) = 0 \tag{S2.1}$$

for a given production function $P_{\text{cue}}(\vec{x},t)$. We approximate the shape of the growing colony at a given time by a disk of radius ρ, and furthermore assume that the density of bacteria within the disk is equal to the carrying capacity $B^* = 1 - \delta/R$, where R is the reproduction rate of the bacteria and δ their death rate, while there are no bacteria outside of the disk. Let r be the distance from the colony centre. Transforming Eq S2.1 to polar coordinates then yields

$$\frac{\partial c(r,t)}{\partial t} = P(r) - dc(r,t) + D\left(\frac{\partial^2 c(r,t)}{\partial r^2} + \frac{1}{r}\frac{\partial c(r,t)}{\partial r}\right) = 0, \tag{S2.2}$$

dropping the subscripts for convenience. Let p be the cue production rate per bacterium. The production function $P(r)$ is then

$$P(r) = \begin{cases} pB^* & \text{if } 0 \leq r \leq \rho \\ 0 & \text{if } r > \rho. \end{cases} \tag{S2.3}$$

We proceed by solving Eq S2.2 for two cases: outside the disk of bacteria ($r > \rho$) and inside the disk ($0 \leq r \leq \rho$). To find the concentration profile outside the disk, we set $P(r) = 0$ and impose the boundary condition $c(\infty) = 0$. The concentration $c(r)$ is then given by

$$c_{\text{out}}(r) = A_1 K_0\left(\sqrt{\tfrac{d}{D}}\,r\right), \tag{S2.4}$$

where $K_n(z)$ is the modified Bessel function of the second kind and A_1 is an arbitrary constant. We ignore any imaginary terms because we are only interested in real solutions. To solve the concentration profile inside the disk, set $P(r) = pB^*$ and remove the boundary condition for $c(\infty)$, because the concentration cannot converge to zero if there is uniform production. Then, the real solution of Eq S2.2 becomes

$$c_{\text{in}}(r) = \frac{pB^*}{d} + A_2 I_0\left(\sqrt{\tfrac{d}{D}}\,r\right), \tag{S2.5}$$

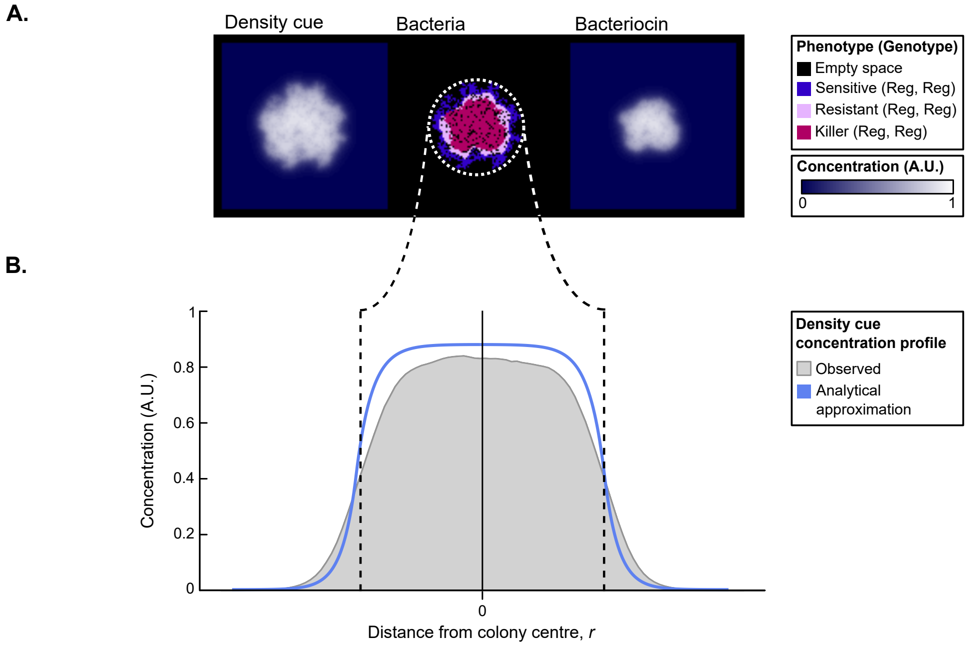

Fig S2.1. Cue concentration profile of a single growing colony (A) Screenshot of a simulation of a single growing colony of regulating killer cells. All cells have toxin production rate $\pi_T = 1$ and response threshold value $\theta = 0.67$, the median response threshold found in serial transfer simulations with default parameters (Table 1 in main text). (B) Analytical approximation of the cue concentration profile (blue) and the actual measured concentration profile (grey).

where $I_n(z)$ is the modified Bessel function of the first kind and A_2 is again an arbitrary constant.

At the perimeter of the colony, $r = \rho$, the solutions for the concentration within and outside the disk should be equal, and since the concentration profile should be smooth, so should their derivatives:

$$c_{\text{out}}(\rho) = c_{\text{in}}(\rho); \qquad \left.\frac{\mathrm{d}c_{\text{out}}}{\mathrm{d}r}\right|_{r=\rho} = \left.\frac{\mathrm{d}c_{\text{in}}}{\mathrm{d}r}\right|_{r=\rho}. \tag{S2.6}$$

From Eq S2.6, the constants A_1 and A_2 can be solved to find the solution for the full concentration profile:

$$c(r) = \begin{cases} \frac{pB^*}{d}\left(1 - \sqrt{\frac{d}{D}}\,\rho\, I_0\big(\sqrt{\frac{d}{D}}\,r\big)\, K_1\big(\sqrt{\frac{d}{D}}\,\rho\big)\right) & \text{if } 0 \le r \le \rho, \\ \frac{pB^*}{\sqrt{dD}}\left(\rho\, I_1\big(\sqrt{\frac{d}{D}}\,\rho\big)\, K_0\big(\sqrt{\frac{d}{D}}\,r\big)\right) & \text{if } r > \rho. \end{cases} \tag{S2.7}$$

In Fig S2.1B $c(r)$ is plotted for the default parameters used in the simulations. The analytical solution provides a good approximation of the observed concentration profile, although it does slightly overestimate the cue concentration in the colony interior. This overestimation occurs because the local carrying capacity decreases when the phenotype of cells switches from sensitive to resistant and toxin producing, but this effect is not incorporated in the analytical approximation.

In the model, bacteria have the size of 1 lattice site and the dimension of distance is lattice site width. Hence, the concentration of density cue molecule experienced by bacteria at the colony edge is given by $c(\rho - \frac{1}{2})$. Furthermore, as the colony size (i.e. ρ) increases, the cue concentration at the colony edge will also increase. To find the maximal concentration experienced by cells on a colony edge, we should therefore consider $c(\rho - \frac{1}{2})$ for $\rho \to \infty$. For the default model parameter values (see Table 1 in main text),

$$\lim_{\rho \to \infty} c(\rho - \tfrac{1}{2}) = 0.484. \tag{S2.8}$$

Hence, the cue concentration sensed by bacteria at the colony edge will be < 0.49.
