# Supplementary figures and images for: Toxin production spontaneously becomes regulated by local cell density in evolving bacterial populations
Source: PLoS Comput Biol. 2019 Aug 30;15(8):e1007333. doi: 10.1371/journal.pcbi.1007333 (PMC6742444; doi:10.1371/journal.pcbi.1007333)

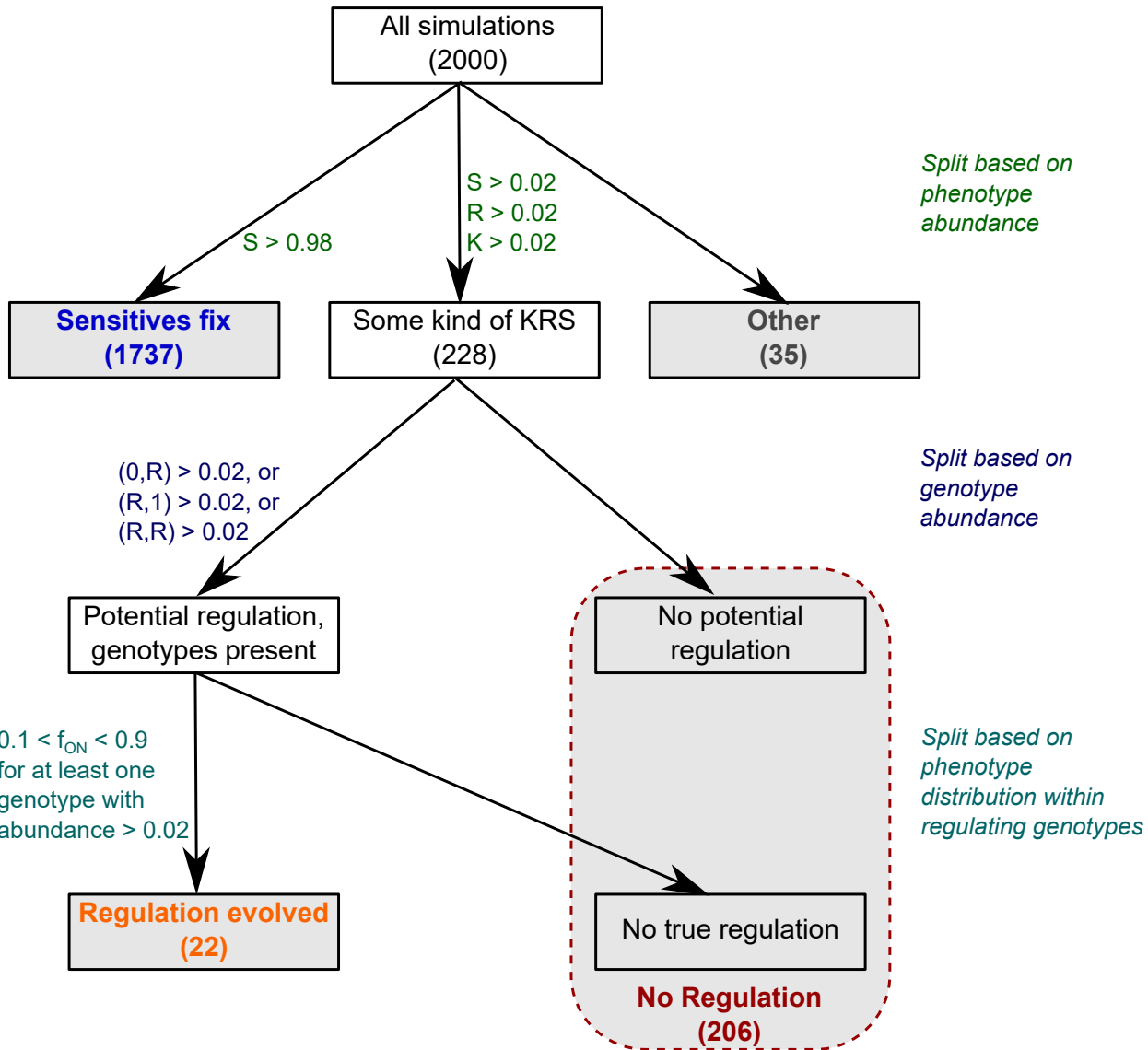

Supplement: S1 Fig — For 2000 different parameter combinations a simulation was run for 400000 time steps, and for each simulation the mean abundance of genotypes and phenotypes in the last 50000 time steps was calculated. Based on these abundance distributions, simulations were classified as showing one of four possible evolutionary outcomes: (i) the sensitive genotype (Off, Off) fixed, (ii) KRS-dynamics arose, no regulation evolved, (iii) KRS-dynamics arose, regulation evolved, and (iv) “other”. This classification was performed in several steps: (1) considering the abundance of different phenotypes in the population (sensitive / resistant / toxin producing), (2) asking if any regulating genotype was present at appreciable abundance (≥ 2% of the population), and (3) asking if such a regulating genotype expressed both of its potential phenotypes (both phenotypes expressed by at least 10% of the regulating cells). This final step ensures that cells identified as regulators indeed switch between phenotypes. (PDF) [file pcbi.1007333.s006.pdf]

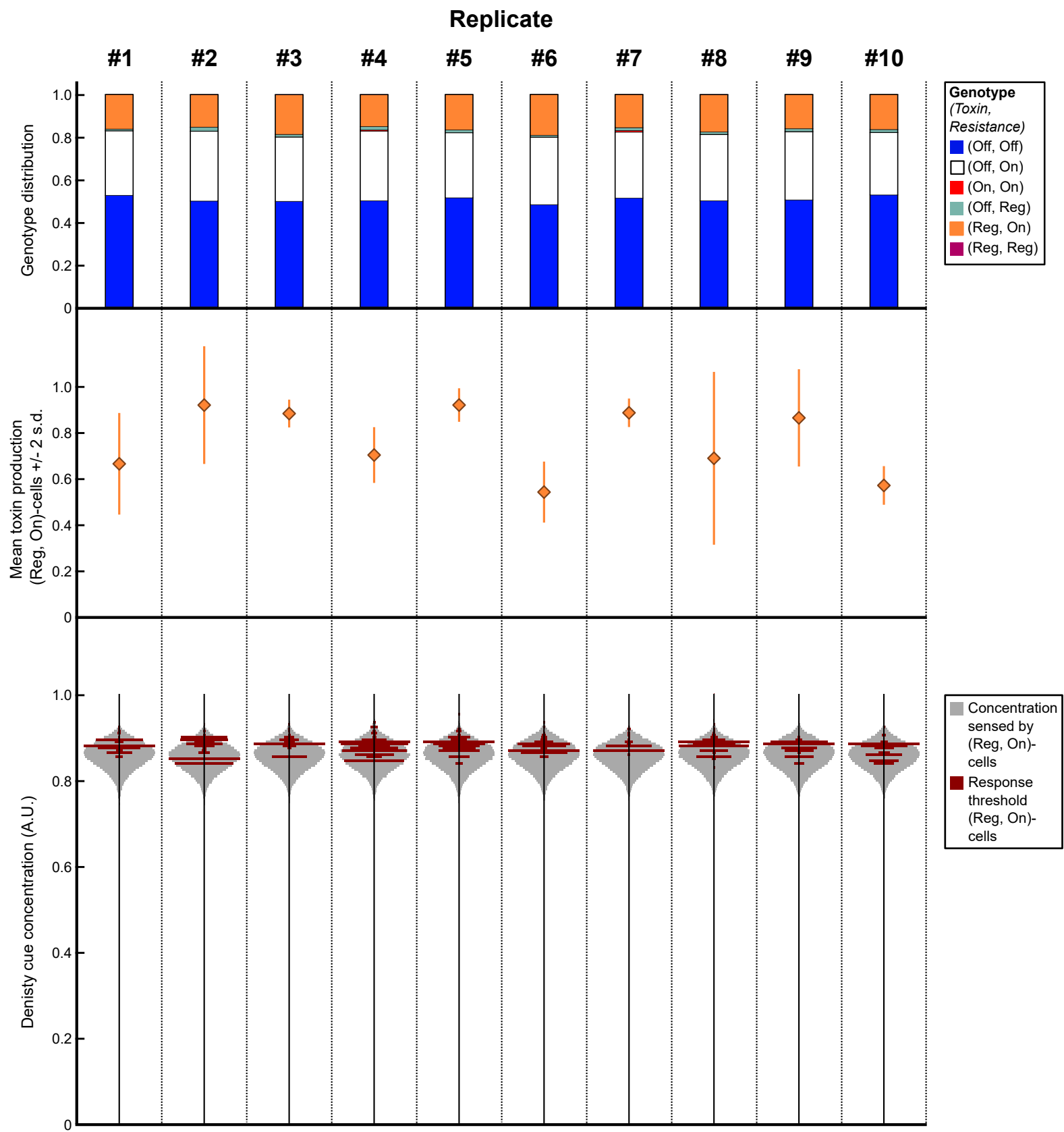

Supplement: S2 Fig — Results of ten independent replicates of the simulation shown in Fig 4. Simulations were run for 400000 time steps, and the genotype distribution was calculated from the mean abundance of genotypes in the last 50000 simulation time steps. In all runs, a KRS-system evolved with regulating (Reg, On)-killer cells, and the genotype distribution at steady state is very consistent over replicates. The evolved toxin production rate did vary somewhat over replicates, but 0.5 < πT < 1.0 in all simulations (middle panel). The distribution of response threshold values θ in the (Reg, On)-cells at the end of the simulation is highly consistent over replicates (bottom panel). (PDF) [file pcbi.1007333.s007.pdf]

**A.**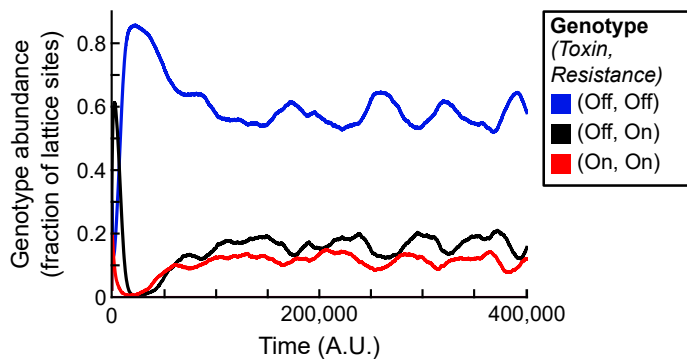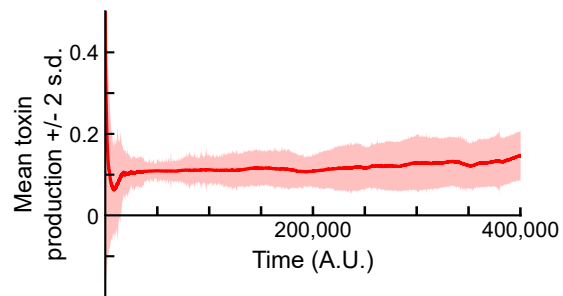**B.**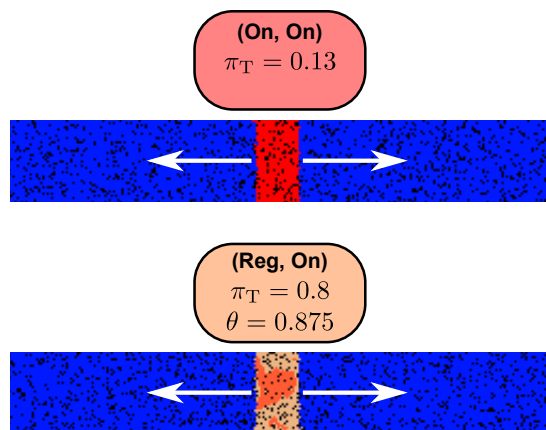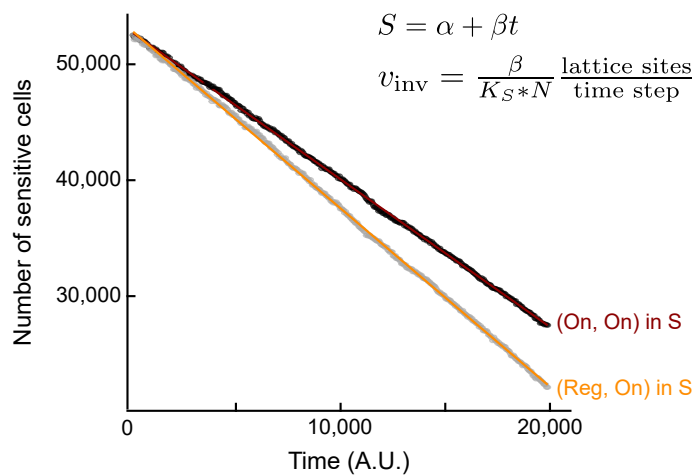**C.**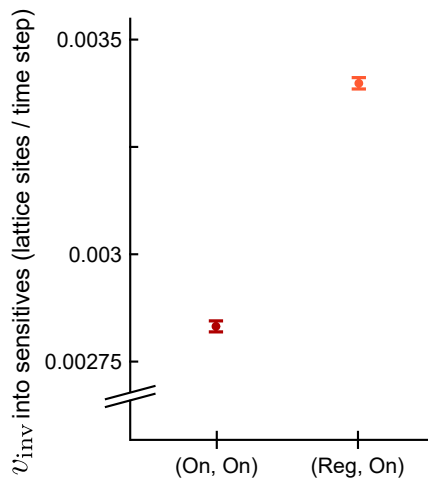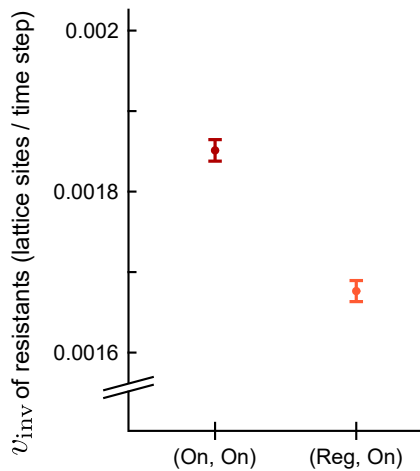

Supplement: S3 Fig — (A) To allow for a fair comparison with the evolved (Reg, On)-cells, constitutive killer cells (genotype (On, On)) were evolved under the same parameter conditions as Fig 4. The example shown here is representative of three replicate runs. (B) Invasion experiments were initialised by placing a 20-cell wide strip of the invading strain in a simulation lattice otherwise filled with the to-be-invaded strain at carrying capacity. The illustration shows the invasion of the (On, On)-strain and the (Reg, On)-strain in a sensitive population; similar experiments were performed for the invasion of a resistant strain in an (On, On)- or (Reg, On)-population. Invasion speed vinv was measured as the decline in the number of sensitives over time, or as the increase of the number of resistant cells over time. (C) Invasion speed of the (Reg, On)-strain into sensitives is higher than the invasion speed of the (On, On)-strain, while invasion speed of the resistant strain is lower in a (Reg, On)-population than in an (On, On)-population. Mean invasion speed ±2 SEM is shown for 10 replicate invasion experiments per combination of invading and invaded strain. (PDF) [file pcbi.1007333.s008.pdf]

**A.**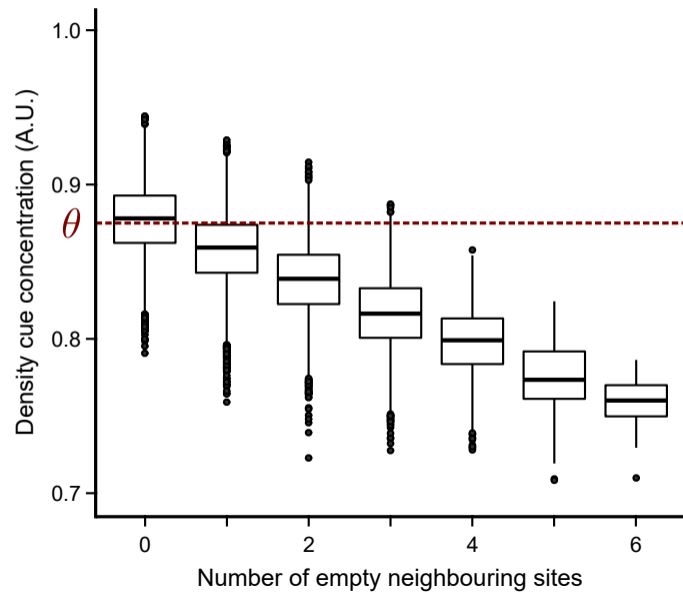**B.**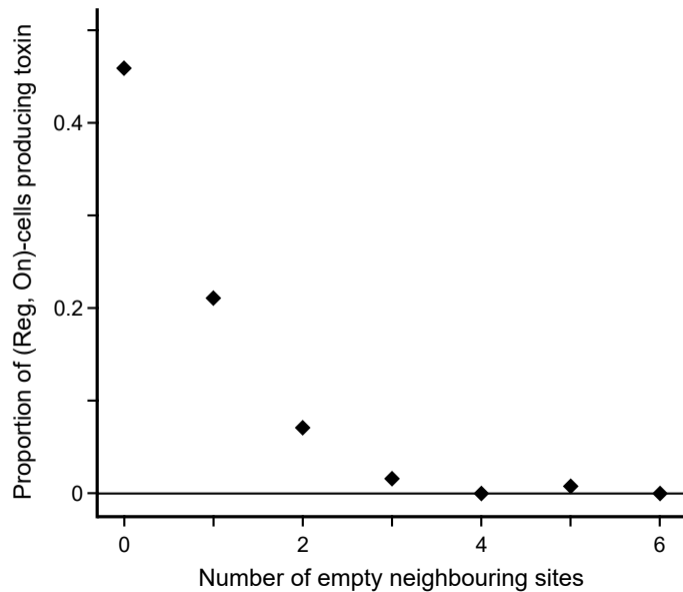

Supplement: S4 Fig — The number of empty neighbouring lattice sites was counted for evolved (Reg, On)-cells at steady state (end of simulation in Fig 4). (A) The concentration of the density cue is highest when cells have no empty neighbours, and decreases with the number of empty neighbours. The mean evolved response threshold value (θ = 0.875) is indicated by a dotted line. Of the cells with no empty neighbours, over half sensed a cue concentration > θ, whereas of the cells with 4 or more empty neighbours, none did. (B) The proportion of cells currently producing toxin as a function of their number of empty neighbours. Around 50% of cells without any empty neighbours produce toxin, while (almost) no cells produce toxin when 3 or more of their neighbouring sites are empty. (PDF) [file pcbi.1007333.s009.pdf]

3 out of 5 replicates

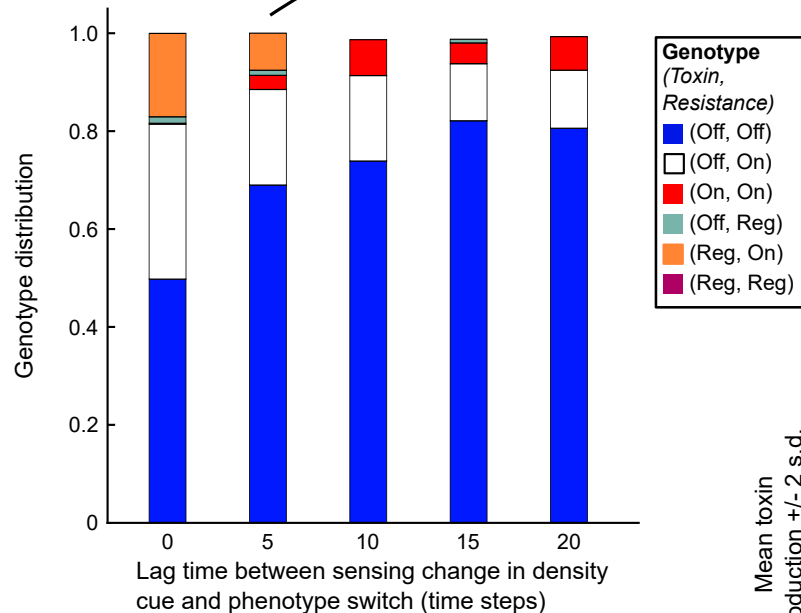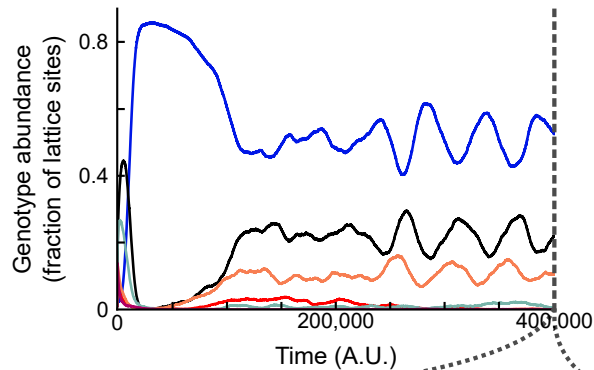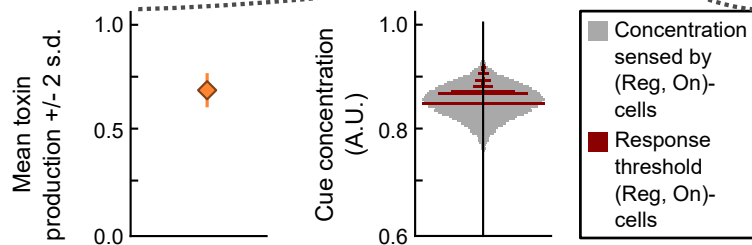

Supplement: S5 Fig — The simulations were repeated for cells that cannot instantaneously adjust their phenotype to the sensed cue concentration, but rather have a lag time between sensing a change in cue concentration and expressing the corresponding phenotype. For each value of this lag time, 5 replicate runs were performed and the genotype distribution was calculated from the mean abundance of genotypes in the last 50000 simulation time steps (evolutionary steady state). For a relatively short lag (5 time steps, which is equivalent to 50% of the minimal bacterial doubling time), regulation still evolved in 3 out of 5 replicates. For longer lag times (≥ 10 time steps), no regulation was found. (PDF) [file pcbi.1007333.s010.pdf]

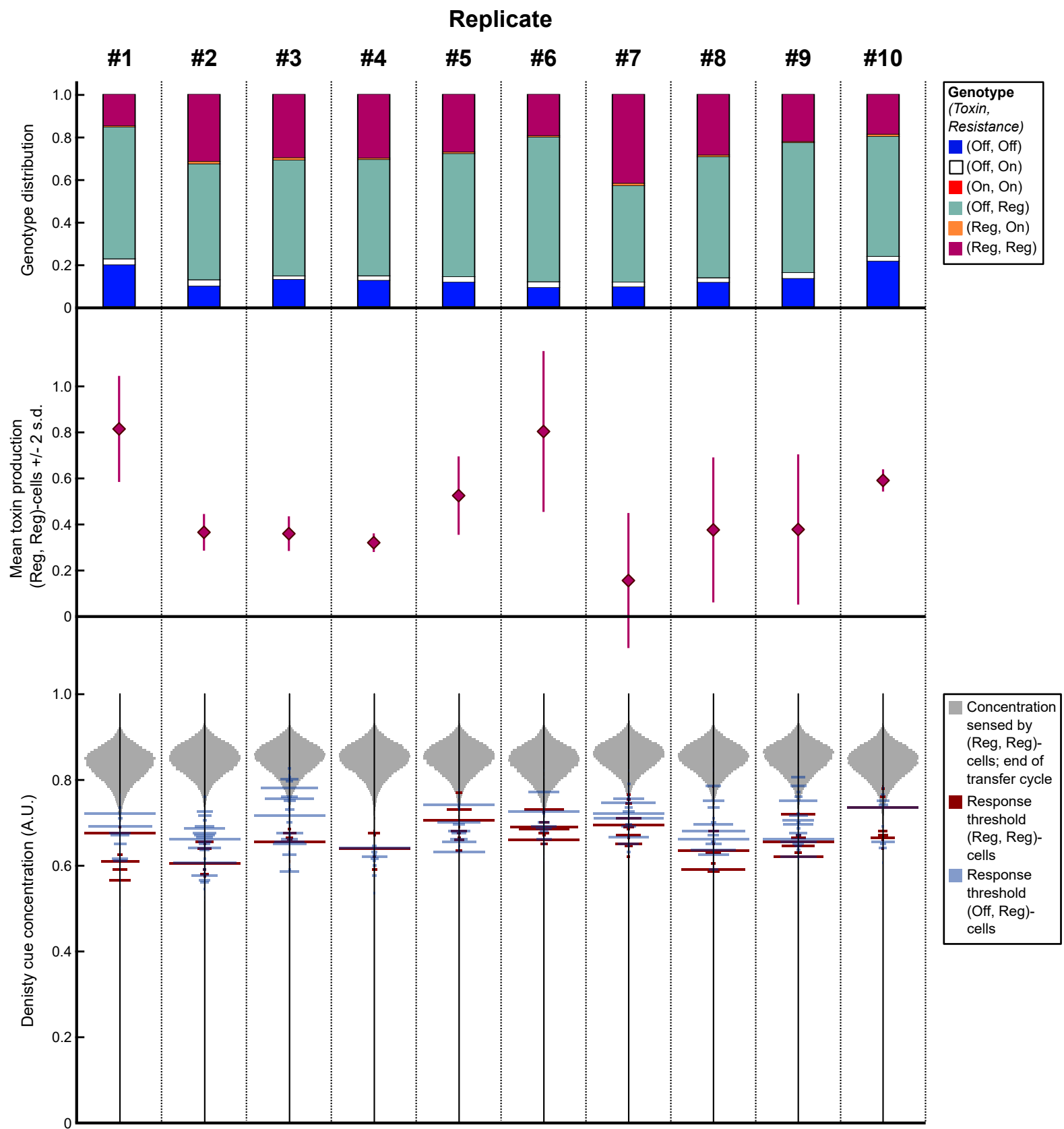

Supplement: S7 Fig — Independent replicate runs of the simulation shown in Fig 5. The top panel shows the genotype abundance profile, which was calculated as the mean proportion of genotypes in the population over the last 50000 time steps of the simulation. The middle and bottom panel show the mean evolved toxin production rate of (Reg, Reg)-cells and the distribution of evolved response threshold values in (Reg, Reg)- and (Off, Reg)-cells at the end of the simulation (Time = 600000). Some quantitative variation exists between replicates, especially in the evolved toxin production rate. However, in all replicates (Off, Off)-, (Off, Reg)- and (Reg, Reg)-cells are selected with similar response threshold values. (PDF) [file pcbi.1007333.s012.pdf]

**A.**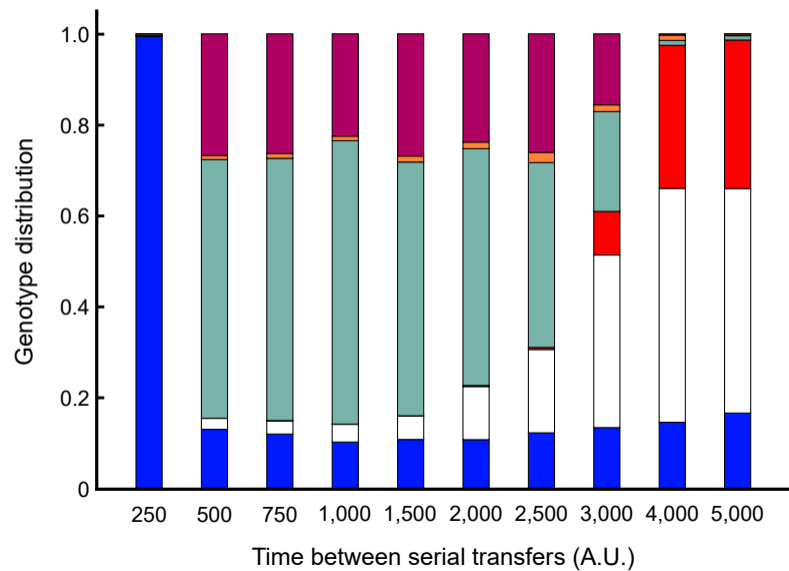**B.**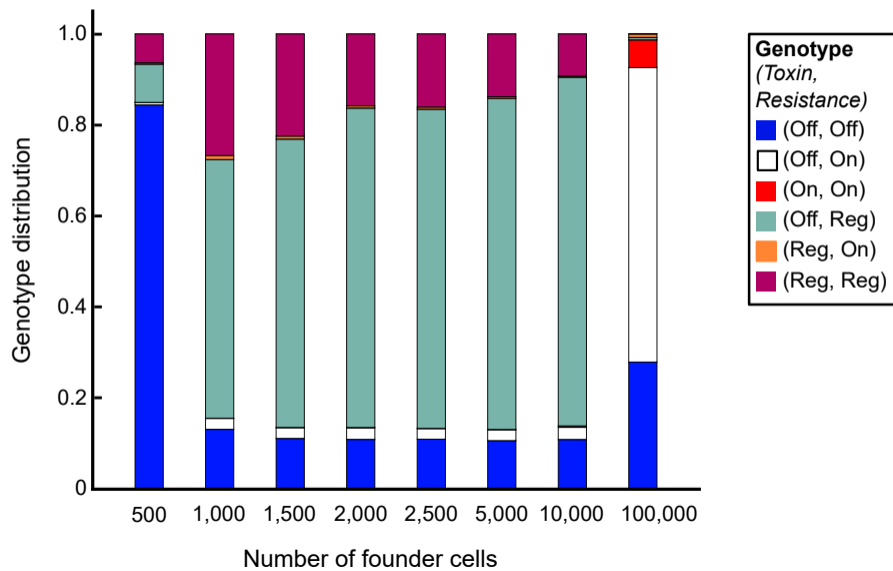

Supplement: S8 Fig — Simulations were performed with parameter conditions as in Fig 5, with the exception of the time between transfers or number of founder cells, which were varied. For each parameter setting, five independent replicate runs were performed. The mean proportion of genotypes over the last 50000 time steps was calculated in the pooled population of all cells in these five replicates. (A) Results of varying the time between transfers. When transfers are very frequent sensitive cells dominate the population, while if transfers are very infrequent non-regulating killer, resistant and sensitive cells are found. However, under a wide range of intermediate transfer intervals regulation readily evolves. (B) Results of varying the number of founder cells. When the population is seeded with very few cells after a transfer, only sensitive cells are selected, while when the number of founder cells is very large a non-regulating KRS-system arises. Again, regulation does evolve for a wide range of intermediate numbers of founder cells. (PDF) [file pcbi.1007333.s013.pdf]

**A.**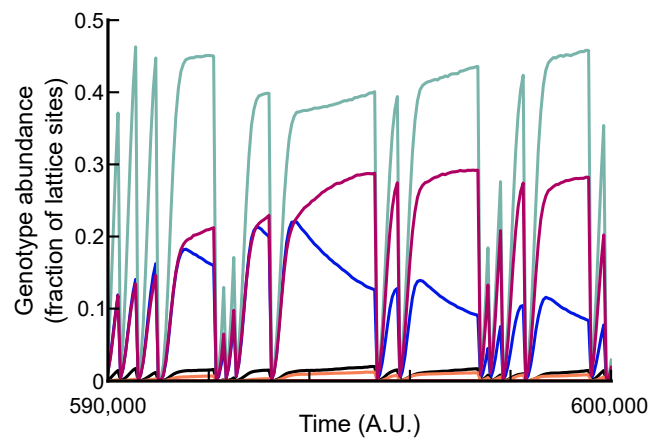**B.**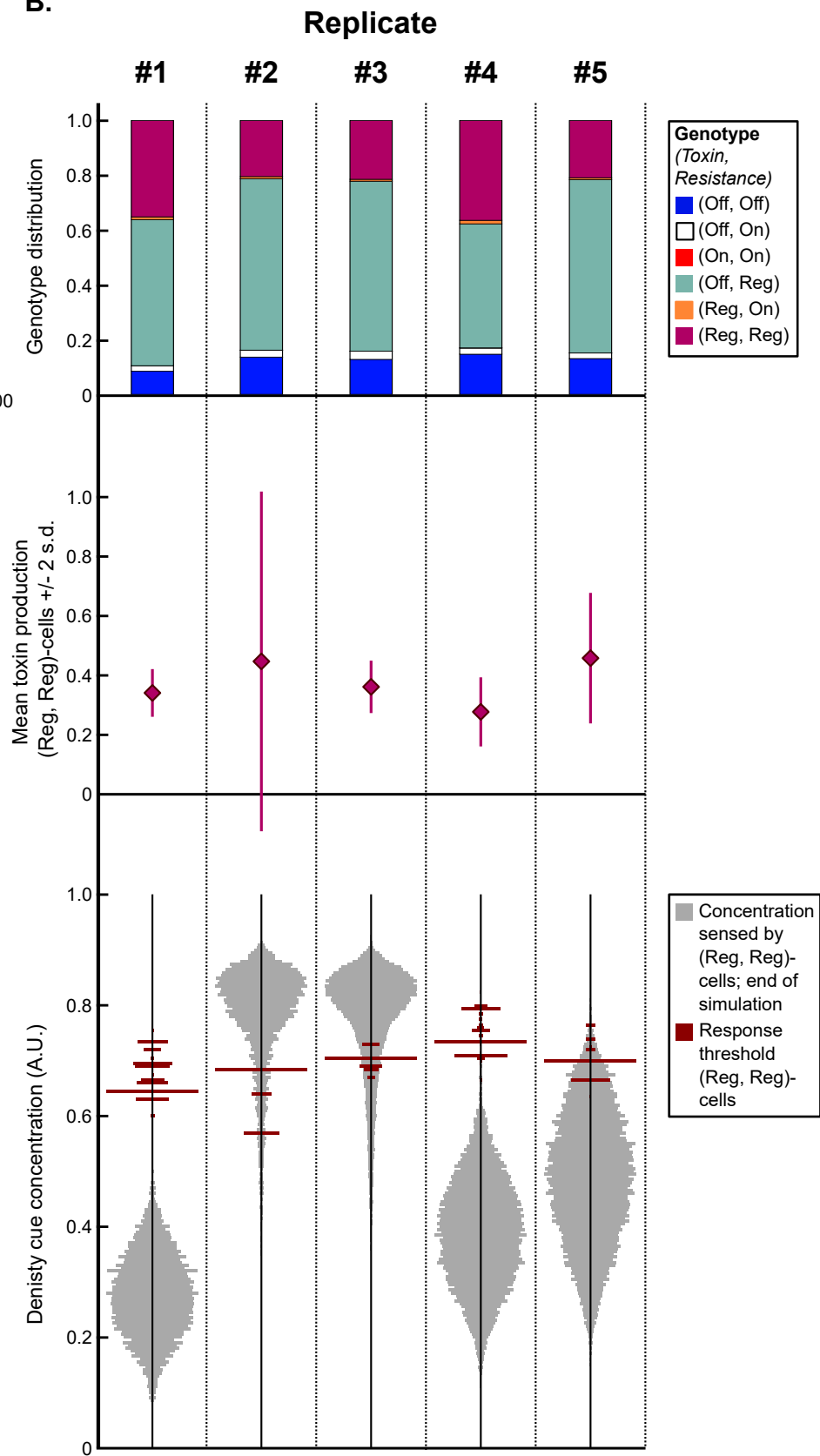

Supplement: S9 Fig — Instead of a fixed time interval between transfers, the length of each transfer cycle was drawn independently from an geometric distribution with mean τtransfer = 500. This way, the mean length of a cycle was kept constant, but transfers now happened at a fixed probability per time step. Other settings were the same as in Fig 5. (A) Population dynamics over a relatively short time interval, illustrating the irregular transfers. (B) Simulation results for five independent replicate simulations. In all five replicates regulation evolved. The results are very similar to the case with regular transfer (Fig 5 and S7 Fig). (PDF) [file pcbi.1007333.s014.pdf]

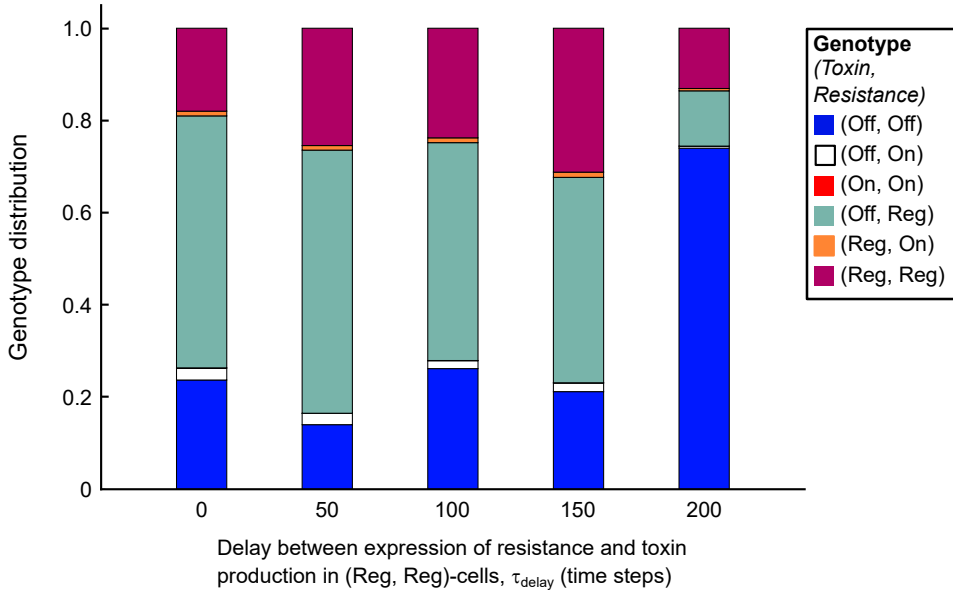

Supplement: S10 Fig — Evolved genotype distribution for varying values of the delay between expression of resistance and toxin production in (Reg, Reg)-cells, τdelay (mean outcome of 5 replicate runs per τdelay value). Regulation of toxin production and resistance (genotype (Reg, Reg)) still evolves when there is no such delay (τdelay = 0 time steps), and when the delay is up to three times higher than the default value (τdelay = 150 time steps). (PDF) [file pcbi.1007333.s015.pdf]

Genotype distribution

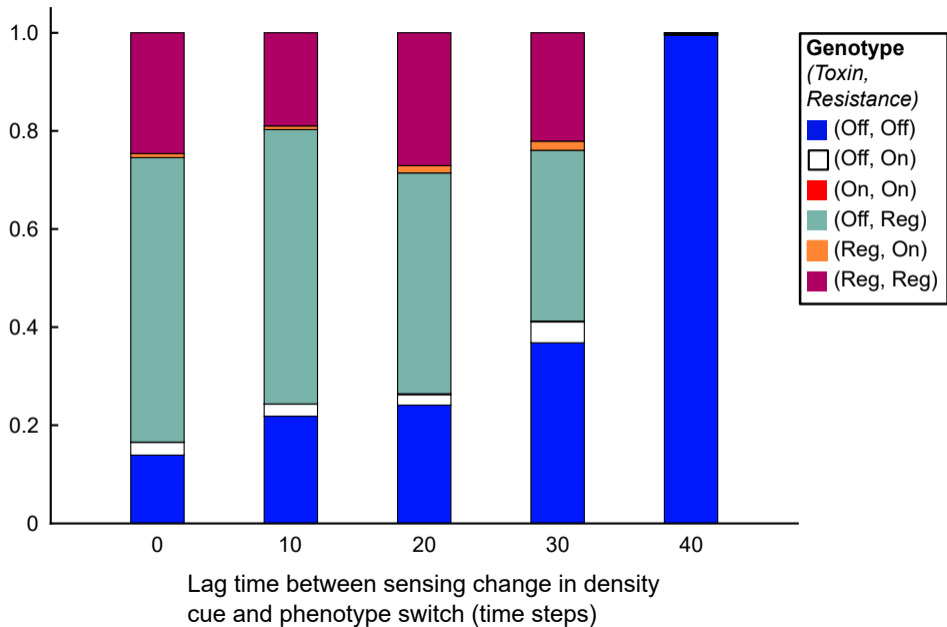

Supplement: S11 Fig — A lag time between cue sensing and phenotype adjustment was implemented as in S5 Fig, and simulations were run for varying values of this lag (5 replicate simulations per lag time value). Under serial transfers, the evolution of regulation is robust to relatively long lag times: regulation still evolved for lag times up to 30 time steps, or 3 bacterial doubling times. (PDF) [file pcbi.1007333.s016.pdf]

**A.**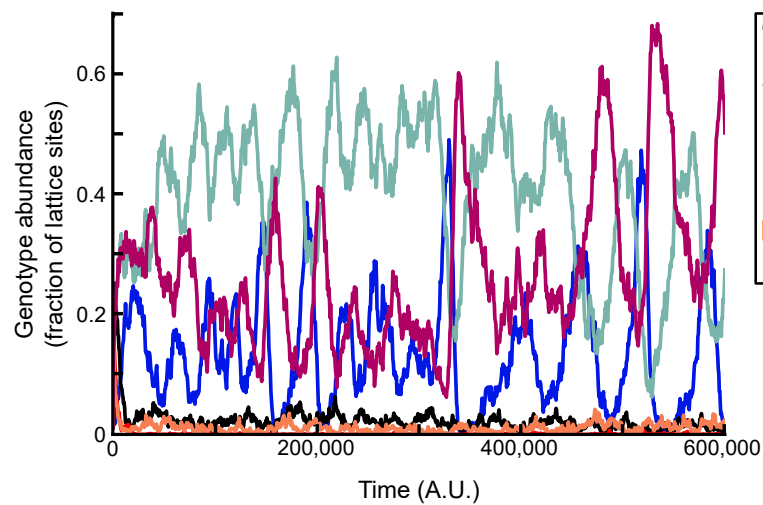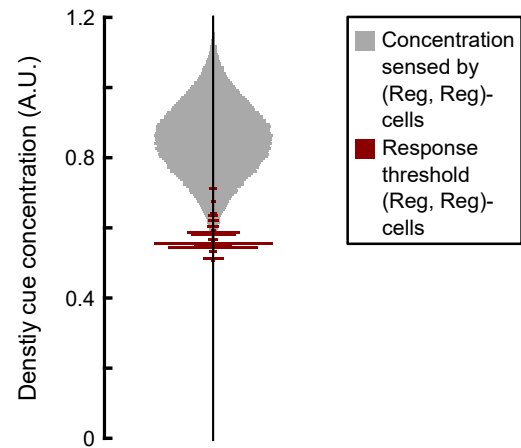**B.**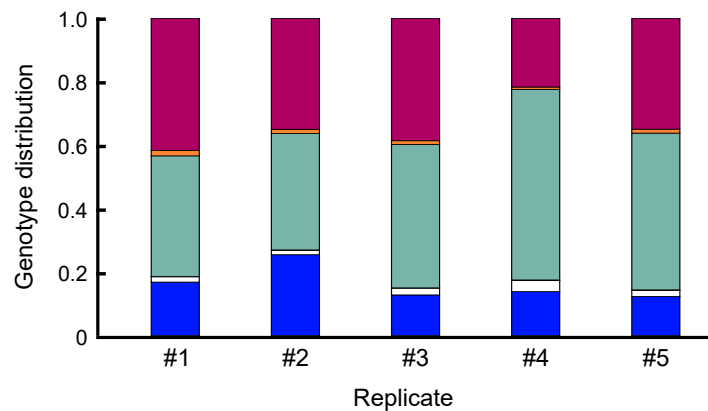

Supplement: S12 Fig — Simulations were run with the same settings as Fig 5, but at each time point at each lattice site a Gaussian noise term (mean μnoise = 0, standard deviation σnoise = 0.1) was added to the local concentration of the density cue. (A) Simulation results of a single, representative run. (B) Summarised results of five replicate runs. Regulation evolved in all five replicate runs. The noise term substantially increased the variation in cue concentrations sensed by cells (grey distribution in right panel of A; note the larger range on the y-axis (c.f., Fig 6)). The evolved response threshold values are however very similar to the values found in the absence of noise (red distribution in right panel of A, compare to Fig 6). (PDF) [file pcbi.1007333.s017.pdf]
