# Supplementary material for: Toxin production spontaneously becomes regulated by local cell density in evolving bacterial populations
Source: PLoS Comput Biol. 2019 Aug 30;15(8):e1007333. doi: 10.1371/journal.pcbi.1007333 (PMC6742444; doi:10.1371/journal.pcbi.1007333)

A.

Constant environment	Serial transfers				
	Sensitives fix	KRS, no regulation	KRS, regulation	Other	*Total*
Sensitives fix	1725	0	3	9	*1737*
KRS, no regulation	134	12	56	4	*206*
KRS, regulation	18	0	4	0	*22*
Other	17	6	5	7	*35*
Total	*1894*	*18*	*68*	*20*	*2000*

B.

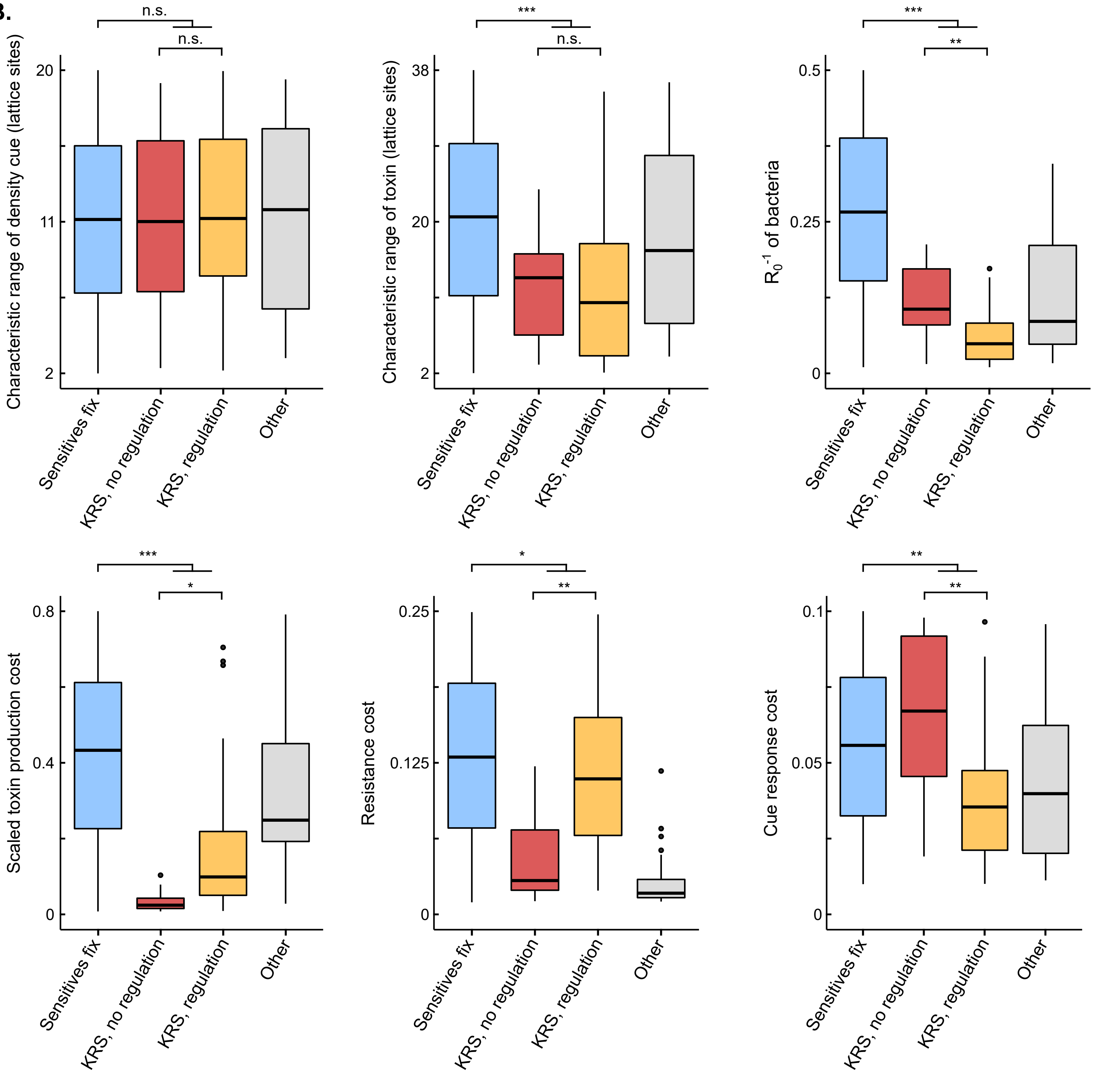

Supplement: S6 Fig — Simulations were run for the same 2000 parameter settings as used for S1 Fig and Fig 3, with the exception that serial transfers were performed once every 500 time steps, reseeding the new population with 1000 founder cells. (A) Table of simulation outcomes, classified as indicated in S1 Fig. Under the serial-transfer regime the sensitive (Off, Off)-phenotype fixes under more parameter conditions than when the simulation is performed in a constant environment, but regulation also evolves more frequently. (B) Parameter conditions in simulations with different outcomes. Significance is shown for 2-sided t-tests with Bonferroni correction for multiple testing: ***: p < 10−10, **: p < 10−3, *: p < 0.05, n.s.: not significant. Toxin production of any type (regulated or non-regulated) is found only when the natural death rate of bacteria is low and phenotypic costs, especially of toxin production, are also low. Among the simulations that resulted in KRS-dynamics, simulations in which regulation evolved have higher toxin and resistance cost and lower response cost than simulations that did not yield regulation. These conditions are similar to the conditions for regulation in the fixed habitat (c.f., Fig 3). (PDF) [file pcbi.1007333.s011.pdf]
